# Detailed reconstruction of the nervous and muscular system of Lobatocerebridae with an evaluation of its annelid affinity

**DOI:** 10.1186/s12862-015-0531-x

**Published:** 2015-12-10

**Authors:** Alexandra Kerbl, Nicolas Bekkouche, Wolfgang Sterrer, Katrine Worsaae

**Affiliations:** Marine Biological Section, Department of Biology, University of Copenhagen, Universitetsparken 4, 1st floor, 2100 Copenhagen E, Denmark; Bermuda Natural History Museum, Flatts, Bermuda, USA

**Keywords:** Nervous system, Musculature, Glandular system, Meiofauna, Annelida, Spiralia, CLSM, Immunohistochemistry, Ultrastructure

## Abstract

**Background:**

The microscopic worm group Lobatocerebridae has been regarded a ‘problematicum’, with the systematic relationship being highly debated until a recent phylogenomic study placed them within annelids (Curr Biol 25: 2000-2006, 2015). To date, a morphological comparison with other spiralian taxa lacks detailed information on the nervous and muscular system, which is here presented for *Lobatocerebrum riegeri* n. sp. based on immunohistochemistry and confocal laser scanning microscopy, supported by TEM and live observations.

**Results:**

The musculature is organized as a grid of longitudinal muscles and transverse muscular ring complexes in the trunk. The rostrum is supplied by longitudinal muscles and only a few transverse muscles. The intraepidermal central nervous system consists of a big, multi-lobed brain, nine major nerve bundles extending anteriorly into the rostrum and two lateral and one median cord extending posteriorly to the anus, connected by five commissures. The glandular epidermis has at least three types of mucus secreting glands and one type of adhesive unicellular glands.

**Conclusions:**

No exclusive “annelid characters” could be found in the neuromuscular system of Lobatocerebridae, except for perhaps the mid-ventral nerve. However, none of the observed structures disputes its position within this group. The neuromuscular and glandular system of *L. riegeri* n. sp. shows similarities to those of meiofaunal annelids such as Dinophilidae and Protodrilidae, yet likewise to Gnathostomulida and catenulid Platyhelminthes, all living in the restrictive interstitial environment among sand grains. It therefore suggests an extreme evolutionary plasticity of annelid nervous and muscular architecture, previously regarded as highly conservative organ systems throughout metazoan evolution.

**Electronic supplementary material:**

The online version of this article (doi:10.1186/s12862-015-0531-x) contains supplementary material, which is available to authorized users.

## Background

Although phylogenomic studies have increased our knowledge of metazoan phylogeny significantly [1–4], a few ‘Problematica’ [5, 6] remain unplaced. Chief among those is the interstitial family Lobatocerebridae, which a recent phylogenetic study based on transcriptomic data positioned within Annelida, as sister group to Sipuncula, albeit with moderate support [7]. This enigmatic group of microscopic, thread-like, fully ciliated animals with glandular epidermis, living interstitially between sand grains in the subtidal sandy sea-floor, was described as its own family, Lobatocerebridae, with one species, *Lobatocerebrum psammicola* [8]. The morphological data available have never indicated a relationship to Sipuncula, although affinities to Annelida as well as to Platyhelminthes have been debated [8, 9]. Due to the ambiguity of the morphological features pointed out by Rieger [8–11], this group was suggested to be its own phylum Lobatocerebromorpha in 1991, alongside annelids, platyhelminthes, molluscs and other spiralians [6, 12]; a status now denied by the recent phylogenomic analyses [7].

*Lobatocerebrum psammicola* was described from the shallow waters off the Coast of North Carolina, USA, based on TEM and LM section series [8–11]. The same articles mention two additional undescribed species from the deep waters off North Carolina and from Eilat, Israel, respectively [8–10]. Additional specimens have been recorded by various authors from marine localities in the Atlantic (for example in Denmark [13], Gran Canaria (Spain) and Elba (Italy, W. Sterrer unpublished), and the Atlantic coast of Panama [7]), but the detailed morphology or taxonomy of these animals (besides *L. psammicola*) has never been investigated. Lobatocerebridae are found in subtidal marine habitats with coarse sand mixed with fine silt, but with limited organic and terrestrial matter. Although found at shallow depths, they are never abundant, and may be mistaken for platyhelminthes, juvenile nemerteans or gnathostomulids, which might explain their understudied nature and lack of additional records. Due to the inaccessibility of material, the explicit descriptions given by R. Rieger in his series of articles [8–11] have remained the only source for systematic and evolutionary discussions for decades [5, 6, 12, 14].

Lobatocerebrids have been described by Rieger [8–10] as having a thin, elongated body with circular cross section and complete ciliation. The epidermis is furthermore interspersed with a high number of unicellular glands. The ventral mouth opening is located one-third of the length from the tip (delineating the rostrum from the trunk), the dorsal male gonopore is positioned two-thirds of the length from the tip, followed by one to several lateral openings of the seminal receptacles in the posterior end of the body and the subterminal dorsal anus. The most prominent and also eponymous character of the animal is the large, multi-lobed brain, which is located anterior to the mouth opening, nearly taking up the entire cross section of the animal. The intraepidermal, ventral nervous system is reported to consist of two lateral nerve cords and two postpharyngeal commissures. The body wall musculature was described as outer longitudinal and inner circular muscles. The animals are simultaneous hermaphrodites [8–10]. Still, none of these morphological characteristics have made a clear classification into or next to one of the existing nominal phyla possible at the end of the 20^th^ century since the identification of common traits has been ambiguous. However, especially Annelida, Gastrotricha, Gnathostomulida, Mollusca, Nemertea, and Platyhelminthes have been discussed as most likely relatives [6, 8, 11, 12]. Details of the epidermis and other characters were examined by Rieger [8–11] with ultrathin (40–70 nm) sections and transmission electron microscopy (TEM), providing information of great ultrastructural detail. However, a detailed cohesive analysis of several organ systems throughout the entire body, including the complete nervous and muscular system mapped with immunostaining and confocal microscopy is still warranted. This will not only enhance our understanding of their morphology but also facilitate a comparison with morphological data on other interstitial groups gathered within the last two decades [15–17].

Both muscular and nervous systems have been assumed to represent rather conserved organ systems when it comes to their general architecture [18]. Annelids, however, have been found to be highly diverse in their morphological characters, and the ancestral states of musculature [19, 20] and nervous system [21] are still debated. The muscular layout in Lobatocerebridae has been described as internal circular and external longitudinal muscles [8, 10], which contradicts the arrangement found in the majority of annelids [22, 23]. However, cases are known where external circular muscles are reduced [24, 25] and several other muscle sets such as transverse, dorsoventral or bracing muscles have been proposed to functionally represent the circular muscles [22]. Nervous system organization has been suggested to be of high systematic importance, revealing synapomorphies of larger clades within e.g., Crustacea [26], which may be undetectable within other organ systems [21, 27, 28]. However, the nervous system in Annelida varies between being intraepidermal to subepidermal [29], in the number of commissures in the brain (2–4, [29]), the number of circumesophageal commissures (1–2, [29]), the number and arrangement of ventral nerve cords (1–7, medio- to lateroventral [15, 21, 29]) and the number and arrangement of commissures in the ventral nervous system (regularly and mid-segmental to irregularly spread along the entire ventral nervous system [15, 21, 29]). Based on the previously available information [8, 10] none of the few characteristics of the musculature or nervous system of Lobatocerebridae could be ascribed to annelids only, since they also show similarities to the pattern described especially from interstitial Gnathostomulida, Plathelminthes, and Mollusca [6, 8–10].

Lobatocerebridae belongs to the meiofauna (animals between 2 mm and 0.06 mm in size [16]), together with exclusively microscopic lineages such as Gastrotricha, Acoelomorpha, Rotifera, Gnathostomulida, Platyhelminthes (except for parasitic forms), Tardigrada, Loricifera, Kinorhyncha, as well as miniaturized forms of macrofaunal lineages such as Annelida, Mollusca and Crustacea [16, 30, 31]. The apparent lack of distinct morphological synapomorphies with other clades, the presence of many autapomorphies, and the inaccessibility of material are the main reasons for why the phylogenetic positioning of these interstitial lineages has been so challenging; and why we only most recently have obtained more information on their evolution [7, 32, 33]. Interstitial fauna (living in the interstices between sand grains) all have a microscopic diameter size and most forms are also categorized as meiofaunal. Besides their small size, these interstitial animals often display simple-looking, worm-like, highly ciliated and glandular, acoelomate bodies with no or few appendages; traits that generally seem to be favored in their confined interstitial environment [16, 34–36]. Several of these seemingly shared traits of interstitial fauna may either have originated as convergent adaptations to their restrictive environment and size, or reflect the recently proposed ancestral meiofaunal condition of Spiralia [7]. Hence, new detailed anatomical investigations of Lobatocerebridae should be evaluated in comparison not only with Annelida, discussing heritage and character evolution, but also with other relevant interstitial metazoans, in order to uncover possible convergent anatomical adaptations to the interstitial space-restricted environment.

The present study will evaluate the recent molecular placement of Lobatocerebridae within Annelida [7], in the light of detailed morphological investigation of nervous, muscular and glandular system with state-of-the-art immunohistochemistry in combination with confocal laser scanning microscopy (CLSM) and transmission electron microscopy (TEM). Hereby, we attempt to unravel and discuss possible resemblances with relevant interstitial spiralians, and whether these common traits may represent annelid synapomorphies, annelid or spiralian plesiomorphies, or convergent adaptations to the space restricted interstitial environment. Furthermore, with the description of *Lobatocerebrum riegeri* n. sp., we are adding another species to this enigmatic, otherwise monotypic group.

## Results

Specimens of *Lobatocerebrum riegeri* n. sp. overall resemble the body plan described by Rieger [8] for *Lobatocerebrum psammicola*. More details of the nervous, muscular and glandular systems could be detected in this study, as described in the following (Figs. 1, 2, 3, 4, 5, 6, 7, 8, 9).

**Fig. 1:**
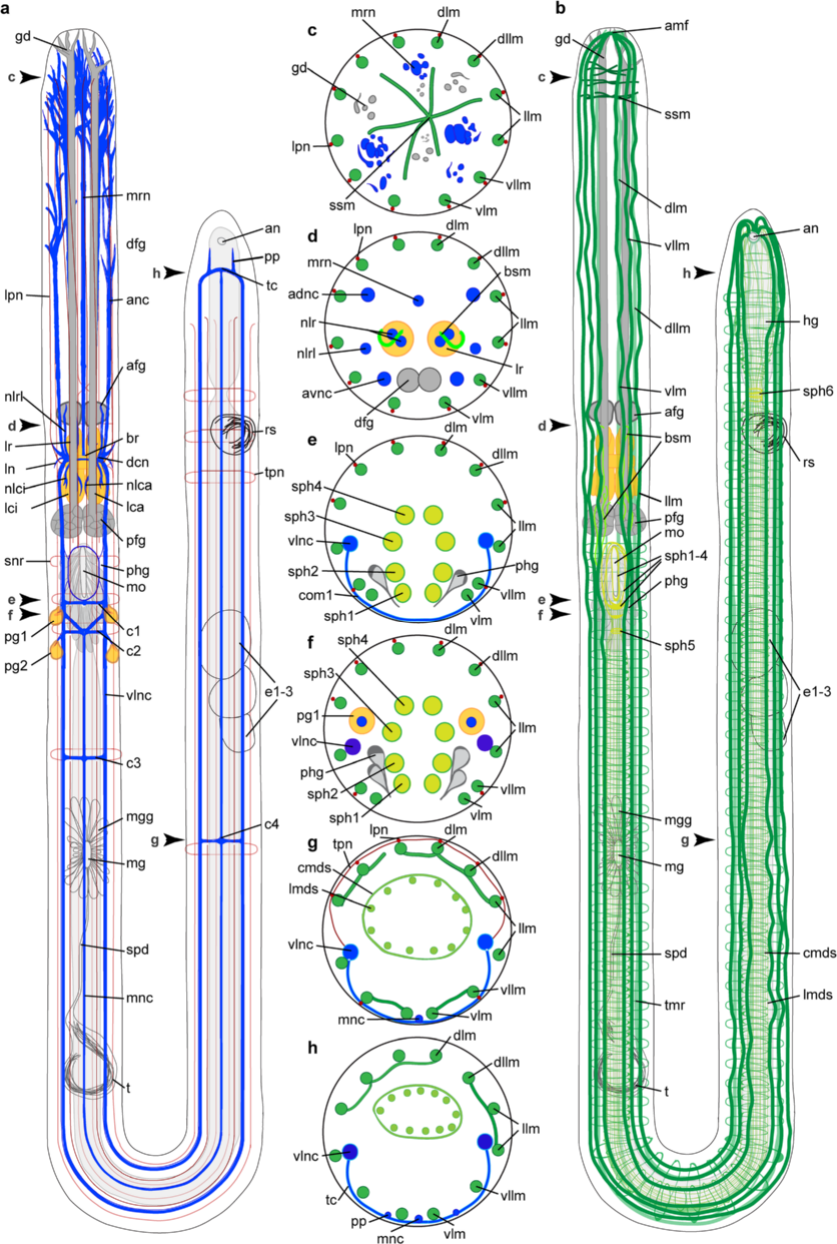
Anatomy of *Lobatocerebrum riegeri* n. sp. as inferred from immunohistochemistry and CLSM. Information is based on all specimens investigated and all antibodies used. **a** Nervous system **b** Musculature, **c**–**g** cross sections in the middle of the rostrum (**c**), at the level of the anterior end of the rostral lobes (**d**), at the level of the first commissure posterior to the pharynx of *L. riegeri* n. sp. (**e**), between the first and the second commissure (**f**), at the level of the forth commissure (**g**) and the level of the subrectal commissure (**h**). Abbreviations: adnc: anterior dorsal nerve cord, afg: anterior frontal gland, amf: anterior point of muscle fusion, an: anus, anc: anterior nerve cord, br: brain, bsm: brain supporting muscle, c1–4: commissures 1–4, cmds: circular muscle of the digestive system, dcn: dorso-anterior commissure of the central neuropil, dfg: frontal gland ducts, dllm: dorsolateral longitudinal muscle, dlm: dorsal longitudinal muscle, e1–3: egg 1–3, gd: opening of the frontal glands, hg: hindgut, lca: major caudal lobe, lci: minor caudal lobe, llm: lateral longitudinal muscle, lmds: longitudinal muscle of the digestive system, ln: lateral nerve, lpn: lateral peripheral nerve, lr: rostral lobe, lrl: lateral rostral lobe, mg: male gonopore, mgg: male gonopore gland, mnc: median nerve cord, mo: mouth opening, mrn: median rostral nerve, nlca: nerve of the major caudal lobe, nlci: nerve of the minor caudal lobe, nlrl: nerve of the lateral rostral lobe, nlr: nerve of the major rostral lobe, pfg: posterior frontal gland, pg1–2: postpharyngeal ganglion 1–2, phg: pharyngeal gland, pp: posterior projection, rs: seminal receptacles, snr: stomatogastric nerve ring, spd: spermioduct, sph1–6: sphincter 1–6, ssm: star-shaped muscle, t: testis, tc: terminal commissure, tmr: transverse muscle ring complex, tpn: transverse ring of the peripheral nervous system, vllm: ventrolateral longitudinal muscle, vlm: ventral longitudinal muscle, vlnc: ventral longitudinal nerve cord

**Fig. 2:**
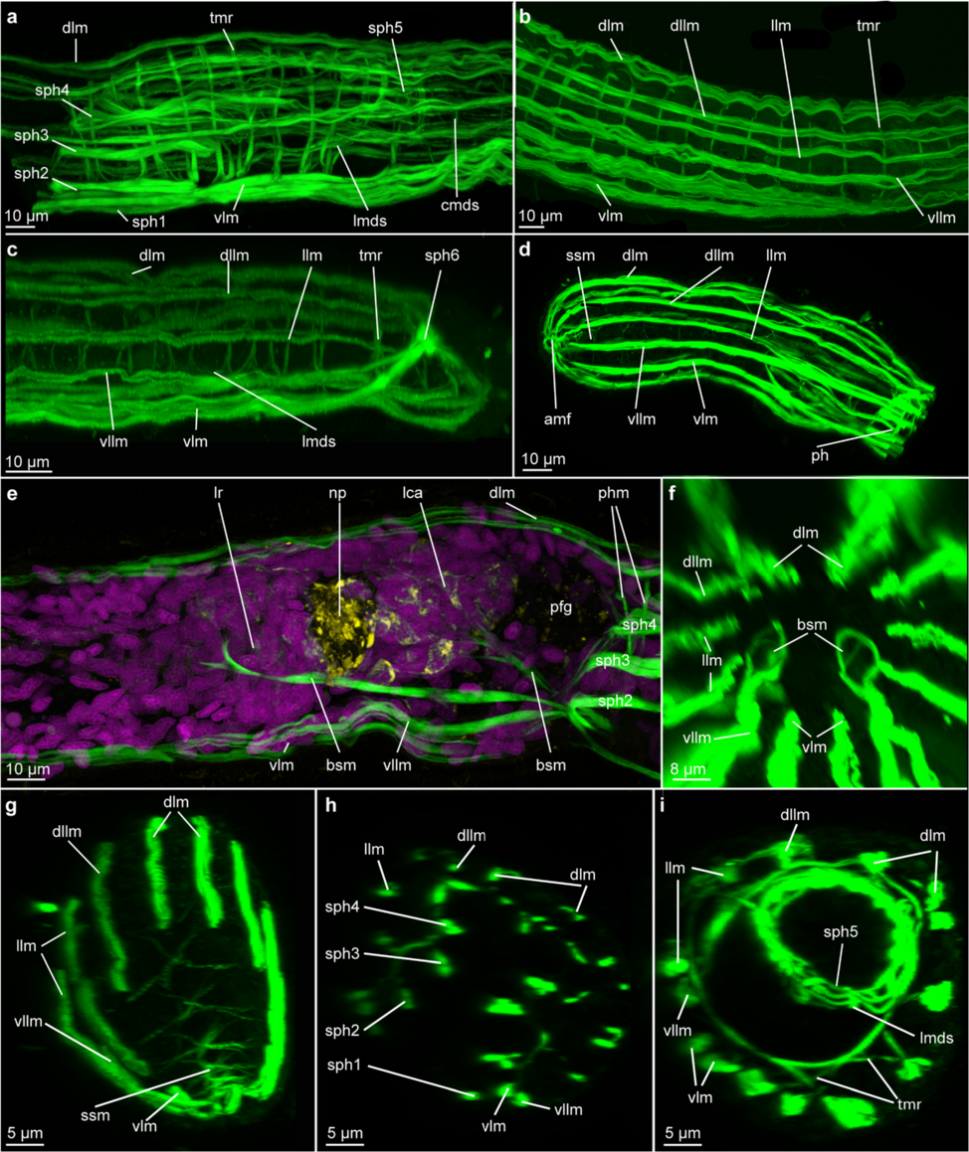
Muscular architecture in *Lobatocerebrum riegeri* n. sp. as seen with CLSM. Musculature (actin-filaments) in green, DAPI in purple, acetylated alpha-tubulin in yellow. **a**, **d**, **g** and **h**) Maximum intensity projections of a juvenile specimen, **b**, **c**, **e**, **f** and **i**) Maximum intensity projections of adult specimens. If not indicated otherwise, anterior is to the left and dorsal is up. **a** Lateral view of the body wall and digestive system musculature in pharyngeal area with sphincters, **b** Lateral view of the body wall musculature in the median region (between the male gonopore and the female ovary), **c** Lateral view of the body wall and digestive system musculature in the posterior tip of the animal, **d** Lateral view of the musculature in the anterior tip, **e** Lateral view of the anterior region of the body wall musculature in a virtually cropped image stack, revealing the brain supporting musculature, **f** Virtual view from inside of the animal towards the anterior tip posterior to the anterior portion of the brain supporting muscles, **g** Virtually cropped view of an anterior tip with star-shaped muscles, **h** Virtual transverse section through the pharynx with sphincters 1–4, **i** Virtual transverse section at the level of sphincter 5. Abbreviations: amf: anterior point of muscle fusion, bsm: brain supporting muscle, cmds: circular muscle of the digestive system, dllm: dorsolateral longitudinal muscle, dlm: dorsal longitudinal muscle, lca: major caudal lobe, llm: lateral longitudinal muscle, lmds: longitudinal muscle of the digestive system, lr: rostral lobe, np: neuropil, pfg: posterior frontal gland, ph: pharynx, sph1–6: sphincter 1–6, ssm: star-shaped muscle, tmr: transverse muscle ring complex, vllm: ventrolateral longitudinal muscle, vlm: ventral longitudinal muscle

**Fig. 3:**
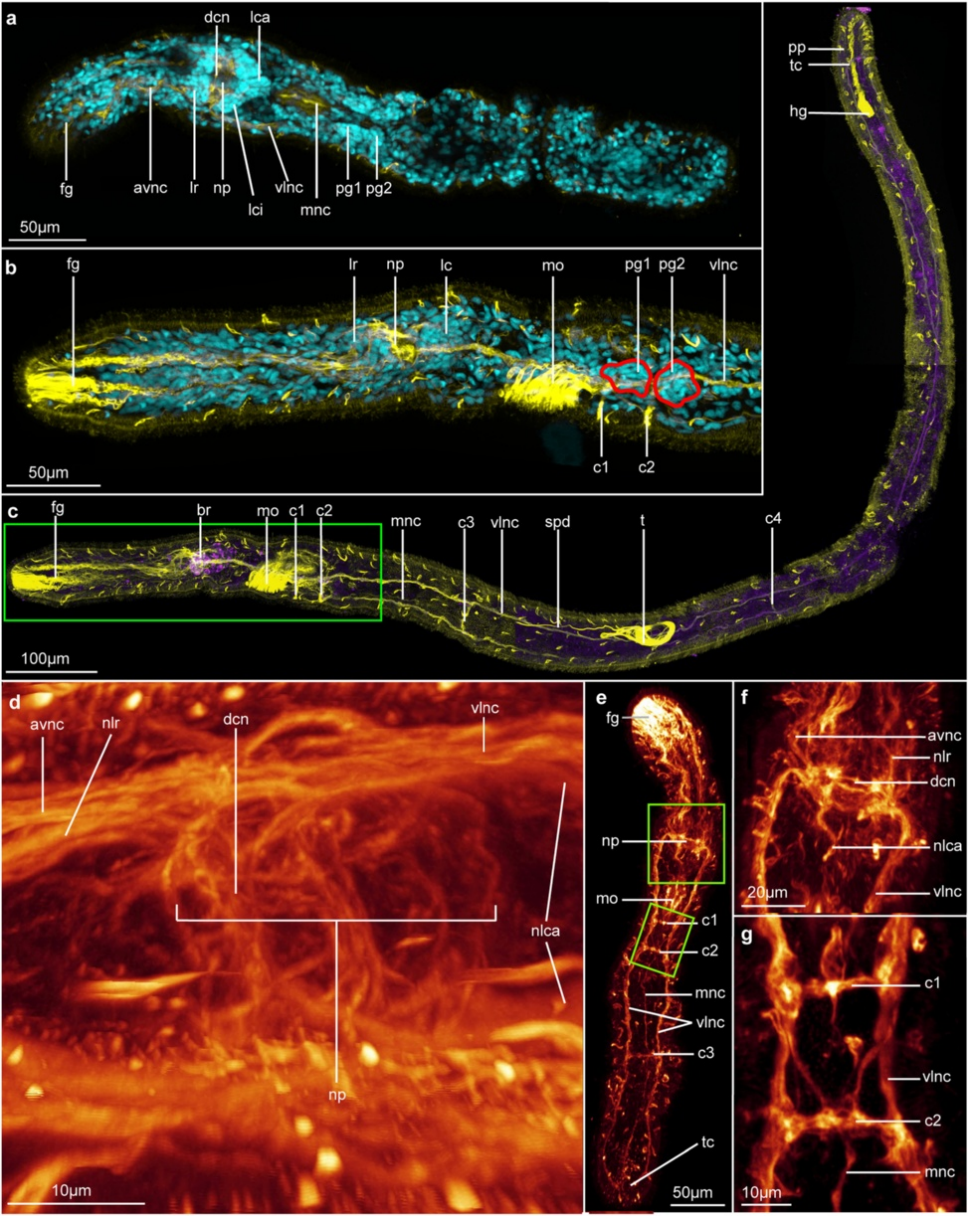
General and detailed organization of the nervous system of *Lobatocerebrum riegeri* n. sp. as seen with CLSM. DAPI in cyan, FMRF in purple and acetylated α-tubulin in yellow or “glow”. All images are maximum intensity projections of a subset of the original image stack on various locations of the body if not specified below **a** Dorsal view of a juvenile specimen, **b** Lateral view of an adult, pharyngeal ganglia outlines in red, details of the same specimen as (**c**), **c** Three different substacks of an adult specimen pieced together for an overview-picture. The specimen is twisted and some portions are laterally oriented and others dorso-ventrally oriented , **d** Dorsal view of the details of the brain, **e** Juvenile showing the general organization of the nervous system. Notice the presence of only three trunk commissures, **f** Dorsal view of the details of the brain in a juvenile. Details of the same specimen as (**e**), **g** Dorsal view of details of the origin of the median nerve cord in a juvenile. Details of the same specimen as (**e**). Abbreviations: avnc: anterior ventral nerve cord, br: brain, c1–4: commissure 1 - 4, dcn: dorso-anterior commissure of the neuropil, fg: frontal gland, hg: hindgut, lc: caudal lobe, lca: major caudal lobe, lci: minor caudal lobe, lr: rostral lobe, mnc: median nerve cord, mo: mouth opening, nlca: nerve of the major caudal lobe, nlr: nerve of the rostral lobe, np: neuropil, pfg: posterior frontal glands, pg1–2: postpharyngeal ganglia 1 - 2, pp: posterior projection, spd: spermioduct, t: testes, tc: terminal commissure, vlnc: posterior ventro lateral nerve cord

**Fig. 4:**
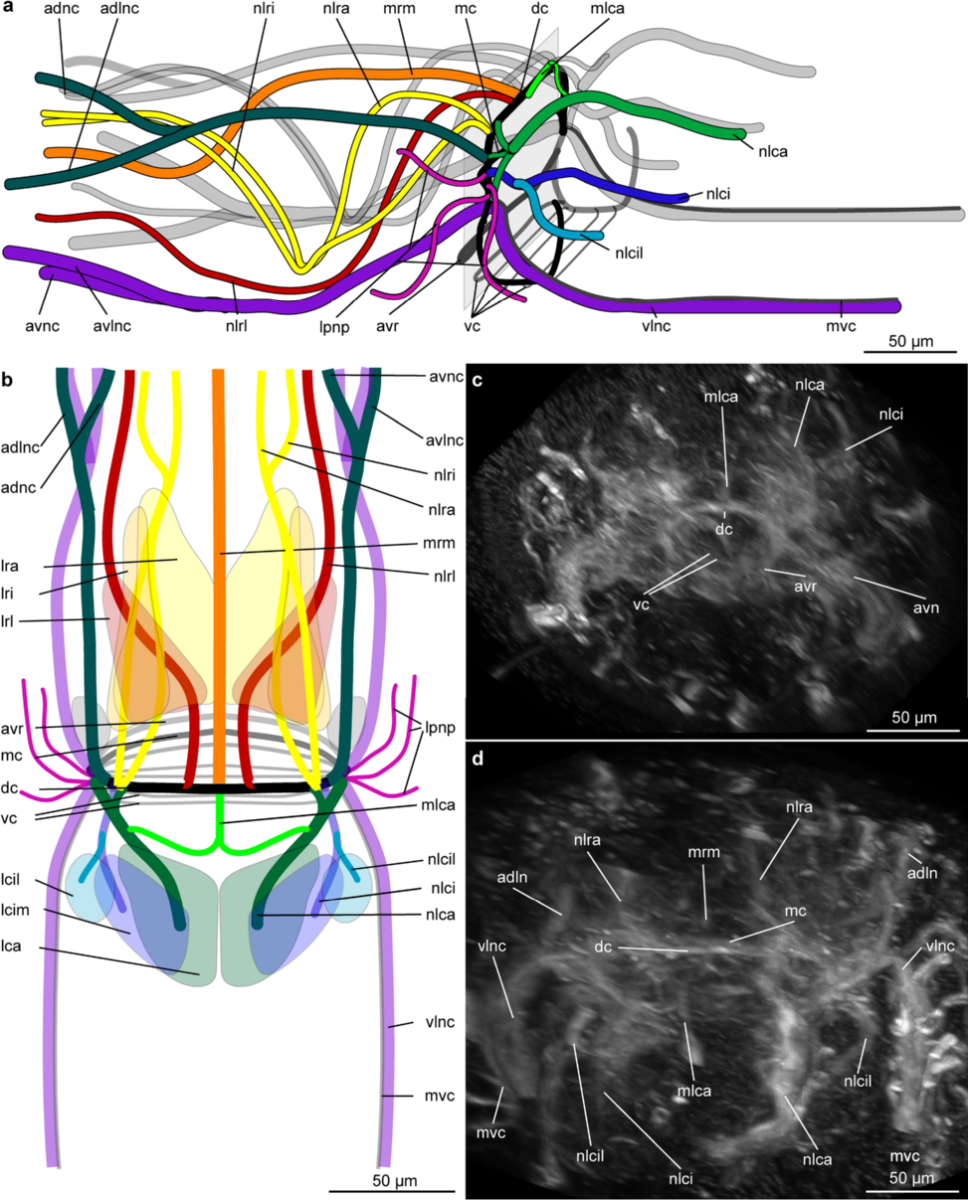
Details of the nerves of the brain of *Lobatocerebrum riegeri* n. sp. as seen with CLSM, acetylated α-tubulin in grey. **a**, **b** Schematic drawings based on confocal stacks, **c**, **d** maximum intensity projections of the original image stack. **a** Brain in dorsolateral view, with the major nerves of the left side colour-coded, the nerves of the right side shaded in grey **b** Dorsal view of the brain with similar colour-coding and indication of the nerves, **c** dorsal view of the central neuropil at the level of the main commissures in the brain; **d** dorsal view of the central neuropil with the major nerve cords as shown in the schematic drawings. Abbreviations: adlnc: anterior dorsolateral nerve cord, adnc: anterior dorsal nerve cord, avc: anterio-ventral commissure of the neuropil, avlnc: anterior ventrolateral nerve cord, avnc: anterior ventral nerve cord, dc: dorsal commissure of the neuropil, lca: major caudal lobe, lcil: lateral minor caudal lobe, lcim: median minor caudal lobe, lpnp: lateral projection of the neuropil, lrl: lateral rostral lobe, lra: major rostral lobe, lri: minor rostral lobe, mc: median commissure of the neuropil, mlca: medial nerve innervating the major caudal lobe, mrm: median rostral nerve, nlca: nerve innervating the major caudal lobe, nlci: nerve innervating the median minor caudal lobe, nlcil: nerve innervating the lateral minor caudal lobe, nlrl: nerve leading through the lateral rostral lobe, nlra: nerve leading through the major rostral lobe, nlri: nerve leading through the minor rostral lobe, mvc: medioventral nerve cord, vlnc: ventral nerve cord, vc: ventral commissure of the neuropil

**Fig. 5:**
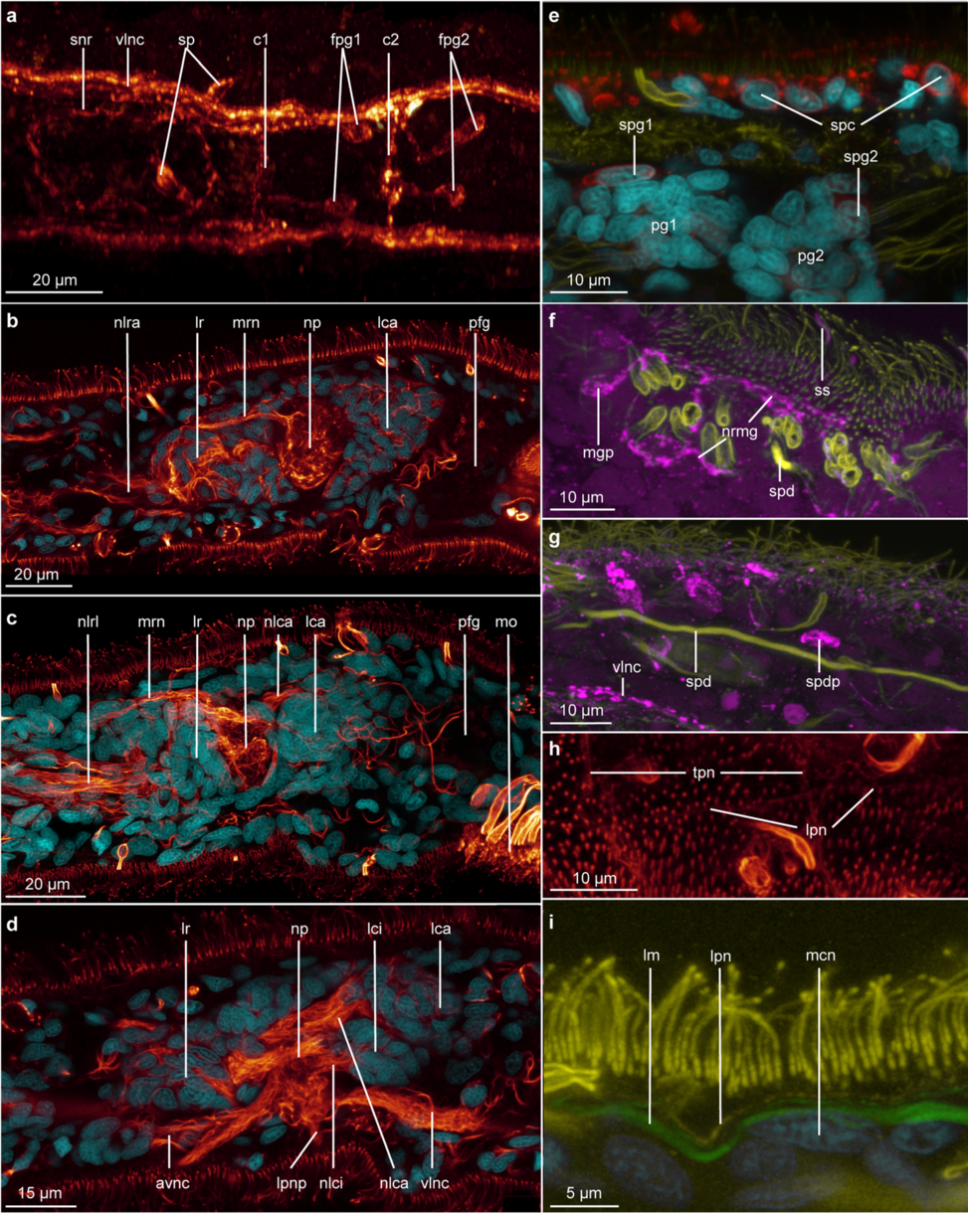
Details of the nervous system of *Lobatocerebrum riegeri* n. sp. as seen with CLSM. DAPI in cyan, serotonin in red, FMRFamide in purple. The use of “glow” depends on the figure and is indicated for each of them. All images are maximum intensity projections of a subset of the original image stack on various locations of the body if not specified below. Anterior is to the left and posterior to the right **a** Dorsal view of the details of the FMRFamidergic nervous system around the pharynx in “glow”. The background noise has been masked to highlight the nervous system. **b**, **c** and **d** Single sagittal sections showing details of the brain. α-tubulin in “glow”, **b**–**d** Virtual sections through the median plane (**b**) the medio-lateral plane (**c**) and the lateral plane (**d**) of the brain, **e** Coronal substack of the animal showing details of the epidermis and the postpharyngeal ganglia (the outside of the animal is on the upper side of the picture), **f** Dorsal view of a sub-stack of the male gonopore (the outside of the animal is on the upper side of the picture), **g** Dorsal view of a sub-stack of the spermioduct, **h** Dorsal view of a sub-stack of epidermis showing the peripheral nervous system with α-tubulin in glow, **i** Sub-stack showing a longitudinal section of the epidermis with details on the peripheral nervous system associated to the musculature (the outside of the animal is on the upper side of the picture). Abbreviations: avnc: anterior ventrolateral nerve cord, c1–2: commissure 1 - 2, fpg1–2: FMRFamidergic perikarya of the postpharyngeal ganglia 1 - 2, lca: major caudal lobe, lci: minor caudal lobes, lm: longitudinal muscle, lpn: longitudinal peripheral nerve, lpnp: lateral projection of the neuropil, lr: rostral lobe, mcn: nuclei of the myocyte, mo: mouth opening, mop: perikaryon associated with the male gonopore, mrn: median rostral nerve, nlca: nerve of the major caudal lobe, nlci: nerve of the minor caudal lobe, nlra: nerve of the major rostral lobe, nlrl: nerve of the lateral rostral lobe, np: neuropil, nrmg: nerve ring around the male gonopore, pfg: posterior frontal glands, pg1–2: postpharyngeal ganglia 1 - 2, snr: stomatogastric nerve ring, sp: perikarya of the stomatogastric nerve ring, spc: serotoninergic cell, spd: spermioduct, spdp: FMRFamidergic perikarya associated to the spermioduct, spg1: serotoninergic perikarya of the postpharyngeal ganglion 1, ss: sensoria, tpn: transverse ring of the peripheral nervous system, vlnc: ventral longitudinal nerve cord

**Fig. 6:**
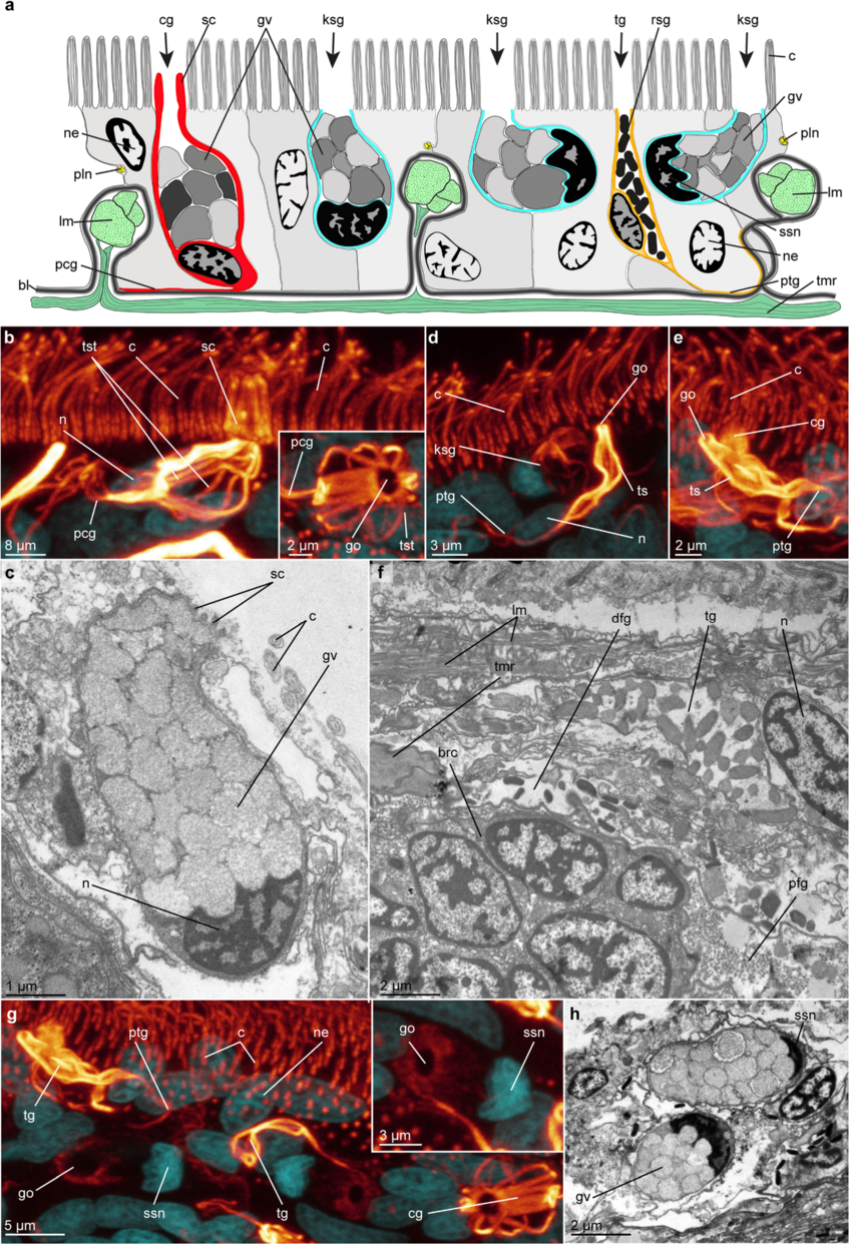
Epidermal glands in *Lobatocerebrum riegeri* n. sp. as seen with CLSM and TEM. DAPI in cyan, acetylated α-tubulin in glow. **b**, **d**–**e**, **g** are maximum intensity projections of a subset of the original image stack on various locations of the body, **c**, **f**, **h** ultrastructural details of the epidermis. **a** Schematic cross section drawing of the epidermis with all three glandular cell types in their approximate abundance, **b** ciliated gland cell with closed circle of shortened cilia (inset with details of the tubular strands in the cellular membrane), **c** Sagittal section of a ciliated gland cell, **d** Tubular gland cell in the epidermis, **e** Tubular gland cell with long projection in the epidermis, **f** Sagittal section through the epidermis of the rostrum, presenting a tubular epidermal gland adjacent to a duct of the posterior frontal gland and the brain, **g** Kidney-shaped glands in the epidermis (inset with details of the glandular opening), **h** Cross section through a kidney-shaped gland. Abbreviations: afg; anterior frontal gland; bl: basal lamina, brc: brain cell, c: cilium, cg: ciliated gland cell, dfg: duct of the frontal gland, go: glandular opening, gv: glandular vesicle, ksg: kidney-shaped gland cell, lm: longitudinal muscle, n: nucleus, ne: nucleus of epidermal cell, pcg: projection of the ciliated gland cell, pfg: posterior frontal gland, pln: peripheral longitudinal nerve, ptg: projection of the tubular gland cell, rsg: rod-shaped granule, sc: shortened cilium, ssn: sickle-shaped nucleus, tg: tubular gland cell, tmr: transverse muscular ring complex, ts: tubulinergic sheath, tst: tubulinergic strand

**Fig. 7:**
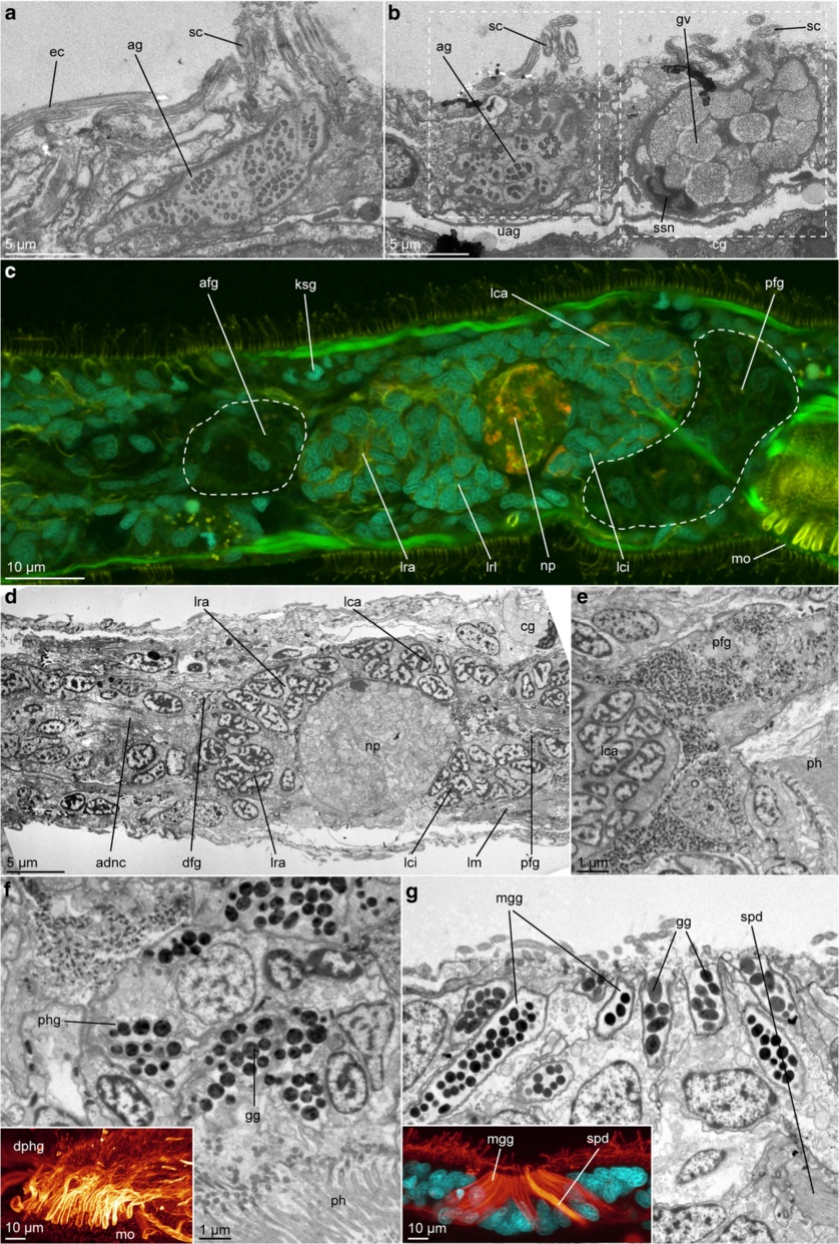
Specific glandular systems in *Lobatocerebrum riegeri* n. sp. as seen with CLSM and TEM. DAPI in cyan, acetylated α-tubulin in glow or yellow, actin-filaments in green. **c**, **g**, **h** are maximum intensity projections of a subset of the original image stack on various locations of the body, **a**, **b**, **d**–**f** ultrastructural details of glandular structures. **a** Sagittal section through the epidermis and an unicellular adhesive gland, **b** Sagittal section through an unicellular adhesive and a ciliated gland in the epidermis, **c** brain and portions of the anterior and posterior frontal glands (indicated by white dashed line), **d** Sagittal section through the anterior tip of the rostrum with ducts of the posterior frontal glands and nerves, **e** Sagittal section through the mouth opening with glandular cells of the posterior frontal gland and the pharyngeal gland, **f** Sagittal section through the pharyngeal region with distal parts of the pharyngeal glands, **g** Distal regions of the ducts of the pharyngeal glands, **h** Glands around the male gonopore. Abbreviations: afg: anterior frontal gland, ag: adhesive granule, cg: ciliated gland cell, dfg: ducts of the frontal gland, dphg: ducts of the pharyngeal gland, ec: cilia of an epidermis-cell, gg: glandular granules, gv: glandular vesicle, ksg: kidney-shaped gland, lca: major caudal lobe, lci: minor caudal lobe, lrl: lateral rostral lobe, lra: major rostral lobe, mo: mouth opening, mg: male gonopore, mgg: male gonopore gland, np: neuropil, pfg: posterior frontal gland, sc: shortened cilium, spd: spermioduct, ssn: sickle-shaped nucleus, uag: adhesive gland cell

**Fig. 8:**
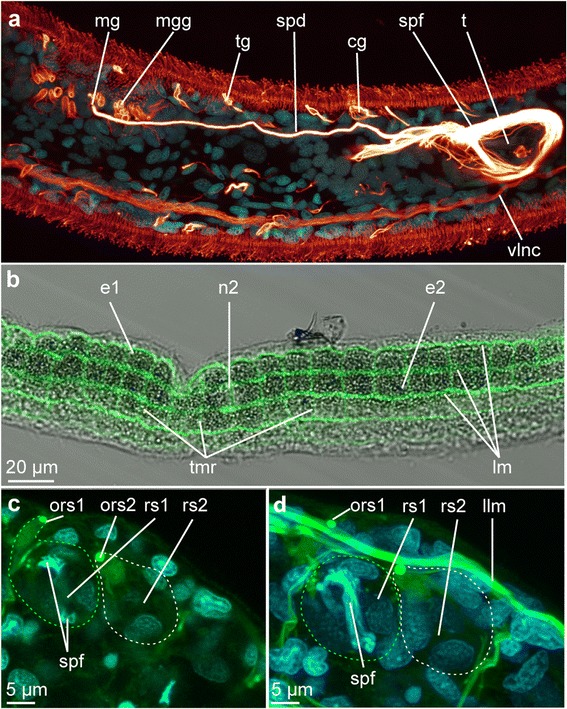
Reproductive organs in *Lobatocerebrum riegeri* n. sp. as seen with CLSM and transmitted light. DAPI in cyan, acetylated α-tubulin in glow, phalloidin in green. All images are maximum intensity projections of a subset of the original image stack. Orientation is anterior to the left and dorsal side up if not indicated otherwise. **a** Testis with spermioduct and glands around the male gonopore, **b** Ovary, **c**–**d** Seminal receptacles at the level of the tips of the sperm filaments **c**) and with bent sperm filaments **(d)**. The contours of the receptacles are traced with dashed lines to facilitate orientation. Abbreviations: cg: ciliated gland, e1–2: egg 1 - 2, llm: lateral longitudinal muscle, lm: longitudinal muscle, mg: male gonopore, mgg: male gonopore glands, n2: nucleus of egg 2, ors1–2: opening of the seminal receptacle 1–2, rs1–2: seminal recepatcle 1–2, spd: spermioduct, spf: sperm filaments, t: testis, tg: tubular gland, tmr: transverse muscular ring complex, vlnc: ventral longitudinal nerve cord

**Fig. 9:**
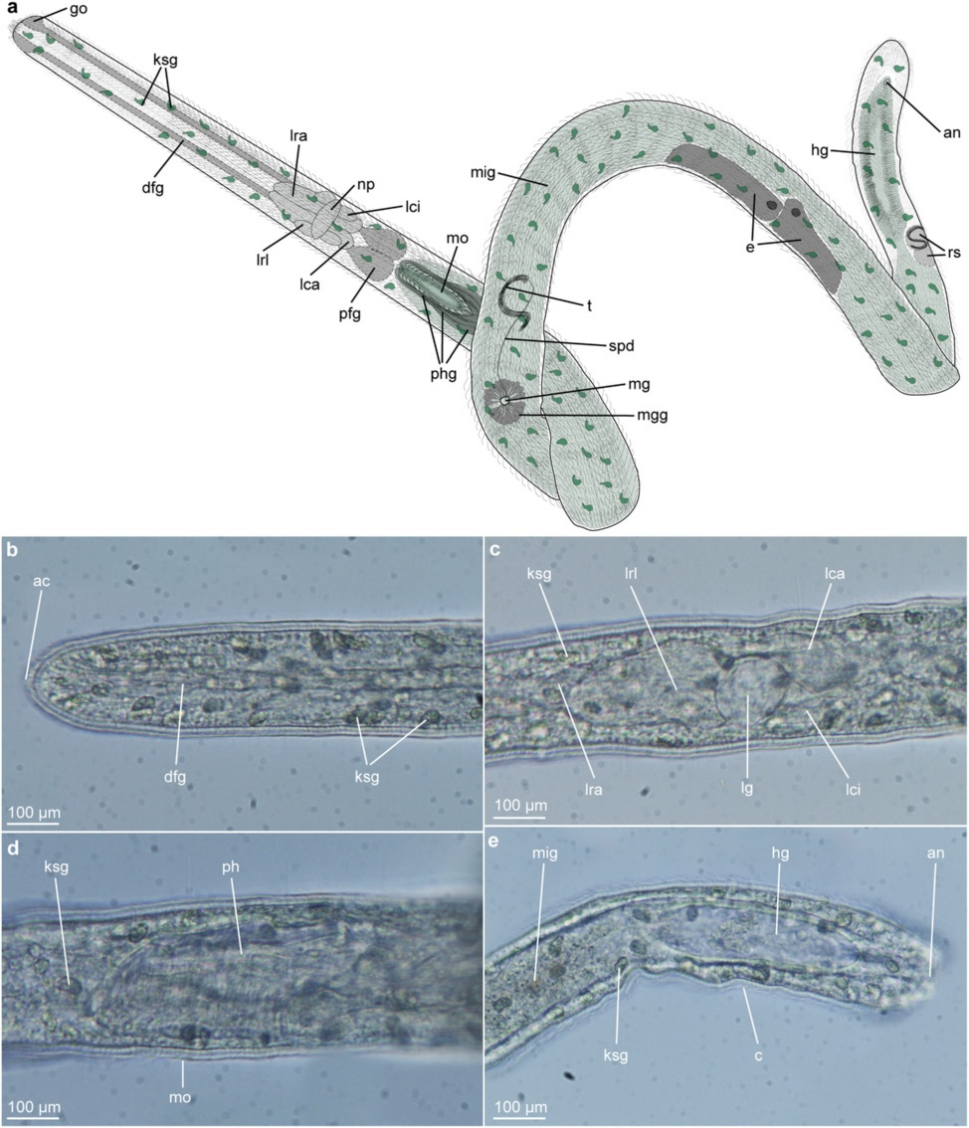
*Lobatocerebrum riegeri* n. sp. Anterior is to the left and dorsal to the upper side of the picture in the light micrographs (**b**–**e**). **a** Partly schematized drawing of an adult *Lobatocerebrum riegeri* n. sp. with the most significant traits emphasized based on light microscopic observation. **b** anterior part of the rostrum with glandular epidermis and frontal gland ducts, **c** brain, **d**, ciliated pharynx and **e** posterior end of the body with midgut-hindgut-transition in lateral view. Abbreviations: ac: anterior cilia, an: anus, c: cilium, dfg: frontal gland ducts, hg: hindgut, go: glandular opening, ksg: kidney-shaped gland, lca: major caudal lobe, lci: minor caudal lobe, lg: lateral ganglion, lra: major rostral lobe, lrl: lateral rostral lobe, mg: male gonopore, mgg: male gonopore gland, mig: midgut, mo: mouth opening, np: neuropil, ph: pharynx, phg: pharyngeal gland, rs: seminal receptacles, spd: spermiduct, t: testis

### Musculature

*Examined in live and preserved specimens in LM; with phalloidin staining in CLSM and ultrathin sections in TEM; Figs.* *1*, *2*.

#### Body wall

##### Longitudinal musculature

As observed by Rieger [8, 10], all muscles of Lobatocerebridae are smooth muscles (confirmed by both CLSM and TEM); no striated musculature was detected in the present study. The longitudinal musculature is organized in six pairs of loose bundles, extending from the rostral tip to the posteriormost end of the body (Figs. 1, 2a–h). Five pairs of these, the dorsal (dlm), dorsolateral (dllm), two pairs of lateral (llm) and one pair of ventrolateral muscle bundles (vllm), lie dorsal to the two prominent ventral nerve cords, whereas the ventral longitudinal muscles are located ventral to those (Fig. 1a–g). Each of these muscle bundles consists of three to five muscle fibres (Fig. 2a–f) and has a diameter of 1.2–2.4 μm (measurements based on: number of specimens (*n*) = 3, region of body (*r*) = 1–4, measurements (*m*) = 5), deeply embedded into the epidermal cells distal to the transverse muscular ring complexes (see below, tmr). The twelve bundles are regularly distributed along the entire body length (spaced 7.2–10.1 μm apart, *n* = 3, *r* = 1–4, *m* = 5, Figs. 1b–g, 2a–e), except around the mouth opening, where the ventralmost pair (vlm) is shifted closer to the adjacent ventrolateral pair (vllm). The male gonopore or the openings of the seminal receptacles do not cause any similar distortions. All twelve longitudinal muscle bundles extend to the posterior end of the body, inserting subterminally around the anus. While the dorsal, dorsolateral and lateral muscles insert directly, the ventrolateral and ventral bundles first trace the epidermis to the terminal end, before bending antero-dorsally and inserting subterminally around the anus (Fig. 2c).

##### Transverse muscular ring complexes

Transverse muscular ring complexes (tmr) are distributed in a regular pattern (spaced 14.5–16.9 μm apart) from the pharynx to the ovary (Fig. 2a), and spaced 6.8–8.9 μm apart posterior of the ovary to the sixth sphincter (*n* = 3, *r* = 2, 3, *m* = 5, Figs. 1, 2b–c). They have previously been misidentified as internal circular musculature [8]. This study, however, could detect that each muscular ring is formed by a series of individual transverse muscle fibres (diameter 0.8–1.3 μm, *n* = 3, *r* = 2, 3, *m* = 5); each of them only spanning the distance between one to three longitudinal bundles (7.6–35.7 μm, *n* = 3, *r* = 2, 3, *m* = 5, Fig. 2j). Up to nine individual transverse fibres are found to constitute one transverse muscular ring complex between all 12 longitudinal muscles (Fig. 2i–j).

Transverse muscles do not form transverse muscular ring complexes in the rostrum, but instead appear as contralateral fibres between longitudinal muscle bundles of opposite sides of the body, hereby creating a star-like structure of individual fibres (star-shaped muscles, ssm, diameter of individual fibres 0.5–1.1 μm, length 10.5–45.2 μm, *n* = 3, *r* = 1, *m* = 5, Fig. 2d, g–h). Their abundance is highest close to the rostral tip (spaced 2.4–5.7 μm apart, *n* = 2, *r* = 1, *m* = 5), where the ducts of the posterior frontal glands are ramifying, and farther separated towards the middle region of the rostrum (spaced 10.3–20.6 μm apart, *n* = 2, *r* = 1, *m* = 5, Fig. 2g–h). The glandular ducts are not muscularized and no closing or constricting mechanism could be detected in this or previous studies [8, 9, 11]. The transverse muscles might therefore be involved in regulating the flow of secretion, in addition to enhancing the flexibility of the rostral tip as observed by behavioral observations (Additional file 1).

##### Additional minor body muscles

Specific musculature is formed around the brain, emerging from the ventral and ventrolateral muscles around the pharynx and extending towards the anterior. The lateral pair of these muscles extends lateroventral to the brain, where the fibres branch off around or into the frontal lobe complex (Figs. 1b, 2e–f). The median pair extends to the caudal lobes, where they branch off into more individual fibres and lead to the major, minor and lateral caudal lobes (Figs. 1b, 2e). Due to the intricate network hereby formed around the anterior and posterior regions of the brain, we suggest these muscles to be a supportive structure for the brain, which is probably necessary due to a lack of other structures securing its position in the rostrum.

#### Intestinal musculature

##### Pharynx

Although lacking a ventral or axial muscle bulb as found in most annelids, the pharynx is still the most prominent muscular structure in the body, showing five sphincter muscles as already defined by Rieger [8] in addition to the longitudinal body and gut musculature. The first four sphincter muscles of the pharynx surround the mouth opening and mouth cavity (sph1-sph4, adapted from Rieger’s sph0-3 [8]), while the fifth sphincter constricts the digestive tract in the transversal plane, as a short esophagus delineating the pharynx from the midgut (sph5, Figs. 1b, 2a, i–j). Sphincters 1–4 consist of two to three fibres each (diameter 0.7–1.6 μm), which are always external to the longitudinal muscles of the digestive tract (Fig. 2j). The fifth sphincter (sph5), however, consists of up to eight thin, serially aligned, muscle fibres (diameter 1.2–1.5 μm, *n* = 3, *r* = 2, *m* = 5). It marks the border to the midgut through an elongated constriction to a diameter of 4.5–4,98 μm when relaxed (*n* = 3, *r* = 2, *m* = 5, Figs. 1b, 2a, j). Additionally, the individual fibres are interwoven with the longitudinal gut muscles, rather than being located externally of these (Fig. 2j).

##### Digestive tract

The intestinal musculature consists of 12 to 16 individual longitudinal fibres (lmds, diameter 0.66–0.74 μm, *n* = 3*, r* = 2, 3, 4, *m* = 5) arranged in equal distance from each other (spaced 1.5–3.1 μm apart, *n* = 3, *r* = 2, 3, 4, *m* = 5), and therefore resembling the muscular pattern of the body wall musculature (Fig. 2a, i). The circular muscles of the digestive system (cmds), however, are arranged external to the longitudinal muscles of the gut (Fig. 2a, j), as is typical for gut musculature. These true circular muscles (as compared to the transverse muscular ring complexes) are very thin (diameter 0.5–0.6 μm, spaced 3.1–5.8 μm apart, *n* = 3, *r* = 2, 3, 4, *m* = 5) and most consistent in the pharyngeal region anterior and posterior to the fifth sphincter. In the posterior part of the body the longitudinal muscle fibres are embraced by the sixth, last sphincter, which consists of two short circular fibres (diameter 1.35–2.1 μm, *n* = 3, *r* = 4, *m* = 3) and constricts the digestive tract to 6.4–6.6 μm when relaxed (*n* = *3*, *r* = *4*, *m* = 4, Fig. 2c). The longitudinal muscles of the digestive system fuse with the longitudinal muscles posterior to this constriction (Fig. 2c).

### Nervous system

*Visualized with acetylated* α*-tubulin IR, serotonin IR, FMRFamide-like IR, DAPI for cell nuclei and CLSM, Figs.* *1*, *2*, *3*, *4*, *5*, *6*, *7*.

The brain in the rostrum of *Lobatocerebrum riegeri* n. sp. is the most conspicuous part of the central nervous system. A series of both anterior rostral and posterior trunk nerve cords emerges from the central neuropil, and some additional nerve bundles are found branching off laterally to the brain (Fig. 4). The brain was described as having one pair of lobes anterior to the neuropil (rostral lobes) and two pairs of lobes (major and minor caudal lobes [8]) posterior to it. However, this study reveals a more complex system of several sublobes both in the anterior and posterior region (Fig. 4a, b). A total of four main commissures in the ventral nervous system (two posterior to the pharynx, one approximately half way between the pharynx and the male gonopore, one anterior to the ovary) are recognized. The anterior two commissures, associated with ganglia, connect the two lateral and the median posterior nerve cords with each other (Figs. 1a, c–g, 4a–c, e). The three longitudinal ventro-posterior cords fuse forming a subrectal commissure. Additionally, peripheral nerves are embedded in the epithelial layer of the animal, forming a grid of longitudinal and semicircular to circular nerves being perpendicular to each other, and being related to the central nervous system.

#### Acetylated α-tubulin-IR

##### Central nervous system: Brain

The brain of *Lobatocerebrum riegeri* n. sp. consists of a large neuropil surrounded by impressive multi-lobed groups of perikarya from where longitudinal nerves extend laterally, anteriorly and posteriorly (Figs. 4, 5). The central neuropil comprises several commissures, which seem to be connecting the two main ventral cords in a pattern possibly resembling the annelid dorsal and ventral root of the circumesophageal commissure. The dorsal, median and ventro-anterior commissures are constituted as well defined nervous bundles, consisting of more than 40 nerve fibres. The ventroposterior commissures cannot always be resolved as individual structures, but form a thin sheath of nervous fibres (Figs. 3d, f, 4).

At least three pairs of characteristic large lobes (or ganglia) are arranged around the central neuropil, namely the paired anterior rostral lobes anterior to the neuropil and the pairs of posterior major and minor caudal lobes (respectively lca and lci, Figs. 3a–b, 4b, 5b–d). The major caudal lobes (lobus caudalis major according to Rieger [8], lca) are located mid-ventrally between the minor caudal lobes (lobus caudalis minor according to Rieger [8], lci, Figs. 3a, 4a–b, 5b–d). The minor caudal lobes seem to be subdivided into a lateral and a median sublobe (lcil and lcim, respectively, Fig. 4b). No postcerebral ganglia as described by Rieger [8] have been found, suggesting that either the lateral sub-lobes of the minor caudal lobes or the lateral ganglia, which were found lateral to the central neuropil, have been mistaken for a postcerebral ganglion by Rieger [8]. The rostral lobes (lobus rostralis according to Rieger [8]) appear to be subdivided into one major (lra) and one minor portion (lri) and one lateral sublobe (lrl, Fig. 4a–b).

Although the nervous network of the neuropil is complex and intricate, some major connections could be reconstructed by means of CLSM. Four paired and one unpaired anteriorly directed rostral nerves all originate independently, but adjacent to each other from the anterolateral parts of the neuropil. In addition, several short nerves project out ventrolaterally from the neuropil for 10 to 20 micrometers (lpnp, Figs. 4a–b, 5d). However, no putative specific structure innervated by them could be identified in that region. The four paired and one unpaired rostral nerves anterior to the brain comprise: 1) One pair of ventrolateral anterior nerve cords extending ventro-laterally from the anterior neuropil (avnc, Figs. 3a, d–f, 5d) as an anterior extension of the posterior main ventral cords. Each of the ventrolateral anterior cords splits into two thinner bundles to innervate the tip and the sides of the rostrum (avnc and avlns, respectively, Fig. 4a–b). 2) One pair of dorsolateral nerves splitting up anteriorly (adnc, adlnc, Fig. 4a–b) originating from the lateral neuropil and possibly connected to the nerve stems of the major caudal lobes. 3) One pair of lateral nerve bundles (nlrl, Fig. 4a–d) originating dorsomedially at the dorsal root commissure but bending ventro-laterally between the lateral and anterior rostral lobes, where after they condense into a thick bundle continuing ventrolaterally throughout the rostrum until they fan out in the anterior end. 4) One loose pair of nerve bundles (nlr, Figs. 3d, 4, 5b–c) originating from the anterolateral neuropil with minor subbundles (nlri and nlra, respectively, Fig. 4a–d) leading medioventrally through the major and minor rostral lobes, joining anteriorly of these, and continuing into the anterior part of the rostrum, before spreading out. 5) One unpaired median nerve (mrm) originating middorsally from the dorsal commissure (dc) between the two rostral lobes and extending dorsally through the entire rostrum, until it eventually splits at the tip to innervate the anterior edge (Figs. 4a–d, 5b–c). The function of such a strong innervation of the rostrum is unknown. However, some nerves connect directly to specific cilia, which are stiff and longer than the locomotory cilia and therefore assumed to have sensory function. Many nerves, however, do not seem to connect to any specific epidermal structures and no multicellular sensory organ could be found. Posterior to the neuropil, two pairs of thick dorso-posterior nerve stems extend posteriorly into the major (nlca) and minor caudal lobes (nlci, Fig. 4a–d); again branching into the two median and lateral parts of the minor lobes (nlci and nlcil, respectively, Fig. 4a–d). The nerve stem of each major caudal lobe is composed of nerves originating from the dorsal commissure (which is suggested to resemble the dorsal commissure of the dorsal root) as well as lateral nerves of the neuropil, the latter being seemingly continuous with the rostral dorsolateral nerves. If truly continuous, this may indicate that the dorsolateral nerves are sensory nerves transferring sensory inputs from the rostrum to be processed in the major caudal lobes.

##### Central nervous system: Ventral cords and commissures

In all specimens investigated, the posterior parts of the ventrolateral nerve cords emerge from the ventrolateral area of the central neuropil and extend to the terminal commissure anterioventral to the anus (pc, Figs. 3c, e). They are located dorsolateral to the third (lateral) muscle bundle, although this position varies slightly throughout the body, with the longitudinal muscles sometimes being so deeply embedded within the epidermis that they become more externally positioned than the nerve cords (Fig. 1e–h). The ventrolateral nerve cords consist of three to four times more fibres than the median nerve and measure 3–4 μm in diameter. The longitudinal ventromedian nerve is located intraepidermally, between the two most ventral longitudinal muscle bundles (mnc, Fig. 3g). It is formed by contralateral projections of the ventrolateral nerve at the level of the first commissure, which fuse in the ventral midline with their counterpart at the level of the second commissure. Hereafter, the median nerve continues posteriorly to insert at the terminal commissure. Two projections from the terminal commissure extend for 10–15 μm dorso-posteriorly (pp, Fig. 3c).

Four trunk commissures are connecting the two ventrolateral nerve cords and the median nerve with each other (c1–4, Figs. 1a, c–h, 3b–c, e, 5a). Each commissure apparently consists of as many nerve fibres as the ventro-lateral cords and measures 3–4 μm in diameter. The anteriormost two commissures are located close to each other posterior to the mouth opening, separated by 20–25 μm (c1, c2, Figs. 1a and 3e, g). Since few of the perikarya of the commissures were showing immunoreactivity against serotonin or FMRFamide, only the large ganglia of the first and second commissures could be detected by a few serotonergic cells and DAPI-staining, here showing densely grouped nuclei (Fig. 5a, e). These ganglia are situated dorsoposterior to the commissures and each consists of 30–40 cells (pg1-2, Figs. 3a, b and 5e). The third commissure is located between the pharynx and the male gonopore, approximately 30-50 μm anterior to the gonopore (c3, Fig. 3c). The fourth commissure (c4, Fig. 3c) is located between the testis and ovary.

Single, presumably sensory, cells are sparsely distributed throughout the epidermis of the entire body, but connect to neither the ventral nerve cords nor the peripheral nerves (ss, Fig. 5f). Normally, they consist of one cell with a single cilium often surrounded by a circle of microvilli (Fig. 5f). There is no correlation between a high abundance of these sensoria and specific organs or body regions.

##### Peripheral nervous system

The peripheral nervous system is embedded in the epidermal cell layer and consists of longitudinal and incomplete circular fibres. These nerves are thinner than the ones of the central nervous system (0.5 μm in diameter) and consist of only very few to individual nerve fibres. The longitudinal peripheral nerves (lpn, Figs. 5h–i) trace the longitudinal muscle bundles throughout the body (lm, Fig. 5i). In the most posterior part of the body, though, they could not be detected with acetylated α-tubulin IR due to the overlaying signal of the central nervous system and the various glands. Their specific origin cannot be assessed, though these thin nerves seem to descend from the central neuropil rather than from the ventrolateral nerve cords.

The incomplete circular nerves (tpn, Fig. 5h) are closely associated with the commissures in the ventral nerve cord, at the level of which they extend from the ventrolateral nerve cords to the dorsal side of the animal. Here, they connect to the longitudinal peripheral nerves exterior to the longitudinal muscle bundles and create a circular connection among these. Additionally and independent of the commissures, one transverse nerve anterior to the pharynx forms an incomplete circle including only lateral and dorsal peripheral longitudinal nerves and three closed rings include all longitudinal peripheral nerves at the level of the seminal receptacles. The latter are set 30–35 μm apart (Fig. 1a). Some additional circular peripheral nerve rings are also found scattered throughout the body. However, they could not be related to any specific structures or reveal a consistent pattern in all specimens investigated.

#### Tyrosinated tubulin-IR

Immunoreactivity of the tyrosinated tubulin-antibody did not reveal any additional structures adding to the pattern already seen with acetylated α-tubulin-IR. On the contrary, the commissure inside the brain as well as the peripheral nerves could not be revealed using this antibody.

#### Serotonin-IR

Serotonin-IR was not only labeling nervous structures, but also glands (uni- and multicellular) and stomach content, where the antibodies most likely got retained between particles or in vesicles (Fig. 5e). However, strong labeling of some but not all epidermal cells could be found, with the IR being located in the entire cytosol, but not in the nucleus, which made them therefore resemble serotonergic perikarya (spc, Fig. 5e). Since there was no connection to the nervous system, they could also be specialized gland or epidermal cells with so-far unknown function.

Serotonin-IR also labels all three longitudinal nerves of the ventral nervous system, with one or two strands inside the thick bundles. This pattern is also present in all commissures, but serotonin-IR cannot be detected in any of the peripheral nerves. In the ganglionic pairs associated with the two pharyngeal commissures, four to five perikarya show serotonin-IR, but do not display any specific arrangement inside the ganglion: They seem to be randomly spread between the other cells (spg1-2, Fig. 5e). Additional perikarya with serotonin-IR are found scarcely along the ventral nerve cord.

#### FMRFamide-like-IR

FMRFamide-like-IR was not consistent between the two specimens investigated. This is mainly due to the rostral glandular structures, which seem to be lying adjacent to the nervous system in *Lobatocerebrum,* and to differences between the studied individuals. Similar to the serotonin - IR described above, the three ventral nerves of the central nervous system, the posterior projection from the terminal commissure (pp, Fig. 5c), as well as the commissures of the central nervous system are revealed using FMRFamide-like-IR (Figs. 3c and 5a). Interestingly, while several nerve fibres in the lateral nerve cords seem to be FMRFamidergic, only one single fibre in the median nerve cord shows this IR, most likely emerging at the level of the pharyngeal commissures. There are no FMRFamidergic perikarya along the ventral nervous system. Only one FMRFamidergic perikaryon in each of the two subpharyngeal ganglia was detected seemingly contributing to the pharyngeal commissure (fpg1-2, Fig. 5a), though its location does not seem to be truly consistent between all specimens investigated.

Possibly as part of the stomatogastric nervous system, two additional pairs of perikarya were revealed dorsal to the mouth and lateral to the pharynx, respectively. Since they are connected ventrally via a thin nerve strand, they seem to constitute the stomatogastric nerve ring described by Rieger ([8], snr, Fig. 5a). Surrounding this structure and disguised by the strong IR of the pharyngeal glands, additional perikarya with very weak FMRFamide-like-IR (sp, Fig. 5a) are found. A further differentiation between the perikarya of the stomatogastric nerve ring and the immune-reactive glands is not possible with any antibody employed in this study.

Though no evidence of the peripheral nervous system could be detected with FMRFamide-like - IR, a FMRFamidergic nerve net is found around the male gonopore. It consists of a thin nerve ring around the male gonopore (nrmg, Fig. 5f) and several individual neurites projecting radially into the ring from their perikarya (mgp, Fig. 5f). Though they are found in all specimens, their number and distribution pattern vary strongly. Additionally, four FMRFamidergic perikarya are distributed scarcely along the spermioduct (spdp, Fig. 5f). No nervous system could be found associated with the ovary or the seminal receptacles.

### Glandular structures

*Studied in LM, with acetylated* α*-tubulin and DAPI staining in CLSM, and in TEM,* Figs. 6, 7*. Acetylated* α*-tubulin-IR of the glandular cell walls* [37] *proved useful to identify and describe several types of glandular cells in the epidermis.*

#### Epidermal glands

Four types of unicellular epidermal glands were distinguished by acetylated α-tubulin-IR and CLSM: a) ciliated glands; b) smooth flask-shaped glands; c) kidney-shaped gland; and d) unicellular adhesive glands.

##### Ciliated glands

The ciliated gland cells (cg, Figs. 6a–c, 7b ) are the largest of the unicellular epidermal glands (diameter 6.9–8.1 μm, length 9.3–11.2 μm, *n* = 3, *r* = 1–4, *m* = 5), distally with a ring formed by shortened stiff cilia around their external opening (sc, diameter 0.6–1.5 μm, *n* = 3, *r* = 1–4, *m* = 5, Fig. 6a, b) and proximally extending into a 30–50 μm long (*n* = 3, *r* = 1–4, *m* = 5), thin tail-region lining the basal membrane. The broad distal region of the gland cells containing the nucleus is located intraepidermally, occasionally alongside the longitudinal muscle bundles, since these are sunken into the epidermal layer (Fig. 6a). The gland cell membranes are lined by twelve to twenty pairwise arranged tubulinergic filaments (tst, *n* = 3, *r* = 1–4, *m* = 5). The cell nucleus has approximately the same size and heterochromatin-content as the nuclei of the surrounding epidermal cells (Fig. 6a–b). The gland cells are packed with non-electron-dense to weakly-electron-dense vesicles (gv, Fig. 6c). They are found scattered throughout the entire body, though they are most abundant in the posterior region, mainly from the midgut-hindgut-transition towards the posterior end of the body. Although the cellular tail region of the cell may tangent a nerve cord, no close connection or direct nervous innervation of the glands, nor indications of muscular control, were found with CLSM or TEM.

These cells most likely resemble the ‘mucous gland type 1’ in *L. psammicola* described by Rieger [8, 10], having a similar characteristic ring of shortened cilia around the opening. This is further corroborated by the similar shape and electron density of the vesicles of these glandular cells [8, 10, 11].

##### Tubular glands

Tubular gland cells (tg) do not have a ciliary ring around their opening, but a continuous lining of acetylated α-tubulin IR in the membrane lining the cell (diameter 1.5–3.2 μm, length 7.8–8.9 μm, *n* = 3, *r* = 1–4, *m* = 5, Fig. 6d, e). They are generally characterized by a slender distal neck-area before the cell widens proximally (Fig. 6a, d, e). However, a few cells with wide distal openings have been found. A long, thin tail extends from the basal part of the cell up to 30 μm along the basal lamina, apparently without connecting to any other structure (Fig. 6a). In contrast to the ciliated glands, the smaller sized tubular gland cells mainly occupy the more distal part of the epidermal layer, distal to the muscle bundles (Fig. 6a). These gland cells are filled with electron-dense, rod-shaped granules (0.8–1.5 μm in length, 0.2–0.5 μm in width, *n* = 3, *r* = 1–4, *m* = 5), which are less densely packed than the vesicles of the adhesive glands (Fig. 6e). They are highly abundant throughout the entire body (10–15 cells per 100 μm body length, *n* = 3, *r* = 1–4, *m* = 5), with the densest distribution in the posterior region of the body.

##### Kidney-shaped glands

Only one glandular cell type (kidney-shaped gland cell, ksg) can be distinguished by the shape of its nucleus: In contrast to all other epidermal cell nuclei, nuclei of kidney shaped gland cells are strictly sickle-shaped (Fig. 6a, g, h) and their chromatin denser than the also “deformed” nuclei of ciliated glands (Fig. 6c). The cell membrane only contains very few tubulinergic elements; yet, dense acetylated α-tubulinergic-IR can be detected around the cell opening (diameter 1.1–1.7 μm, *n* = 3, *r* = 1–4, *m* = 5) and at its base. The overall appearance of the cell is characteristically kidney-shaped (diameter 3.3–4.7 μm, length 6.9–7.8 μm, *n* = 3, *r* = 4, *m* = 5, Fig. 6e). Kidney-shaped gland cells are mainly found in the distal part of the epidermal layer similar to the tubular gland cells (Fig. 6a). However, the basalmost part of the cell, which contains the nucleus, can also be found close to or even internal to the longitudinal muscle bundles (Fig. 6a, g). These glandular cells are most likely imparting the greenish speckled appearance of the animals in live observations (Additional file 1) due to the refractive index of their content, which consists of non- to weakly-electron dense and tightly packed vesicles (diameter 0.6–1.2, *n* = 3, *r* = 1–4, *m* = 5, Fig. 6h). In contrast to the ciliated gland cells, the vesicles of the kidney-shaped gland cells are less homogenous in the electron-density of their content, and denser in their packing, possibly causing the sickle-shape of the nucleus.

##### Unicellular adhesive glands

The unicellular adhesive glands are characterized by a ring of shortened cilia around the opening, which was suggested to facilitate mechanical loosening from the substrate instead of a second enzymatic gland with releasing function [8, 10] and therefore morphologically resembles the ciliated glands though their content and function differ (Fig. 7a, b). Their secretion is granular, but shows a characteristic structure with an inner, electron-dense area in a non-electron-dense oval structure (Fig. 7a, b). Different to the adhesive glands described in *L. psammicola*, the glands of *L. riegeri* n. sp. do not have linear electron-dense structures in the middle of the individual granules, but instead linearly arranged electron-dense dots (Fig. 7a, b). Contrary to the abundance and distribution pattern of the other epidermal glands cells mentioned above, adhesive gland cells are restricted to the ventral surface of the body in lower numbers (1–5 cells per 100 μm ventral body length, *n* = 3, *r* = 1–4, *m* = 5).

#### Frontal glands

The main body of the paired posterior frontal glands (pfg) is found posterior to the brain and anterior to the pharyngeal region (Figs. 5b, c , 7c). This part of the glands is difficult to detect with any of the antibodies described above, but can be found combining the lack of DAPI-signal with overexposed phalloidin-signal to detect cell membranes and nuclei of voluminous cells in a large lobular structure posterior to the brain lobes (Fig. 7c). The glandular nuclei are slightly larger than the ones of the brain (diameter 4.3–5.7 μm × 1.4–2.5 μm, *n* = 3, *r* = 1, *m* = 5). While the gland body itself is inconspicuous in CLSM, its long ducts, which are leading ventroanterior of the brain to the tip of the rostrum, are showing distinct acetylated α-tubulin-IR (Fig. 3a–c, e, 7d). Posteriorly the ducts are straight and grouped into two bundles; anteriorly they ramify into a fan of duct openings framing the anterior edge (Figs. 1a, 3b, c, e). Ramifying longitudinal nerves are found accompanying these in the rostrum but possible nervous innervation of the frontal glands could not be resolved. The cellular content of the posterior frontal glands consist of very small (diameter 0,2–0,3 μm, *n* = 1, *r* = 1, *m* = 10) spherical, electron-dense granules, which seem to increase in diameter towards the anterior tip of the animal and the opening of the duct (Fig. 7d, e). This glandular content can clearly be distinguished by their shape from the content of the epidermal cells described above (big vesicles) and the granules of the anterior frontal glands (rod-shaped granules, Figs. 6a, c, f, h, 7a, b, d–f).

An additional, smaller pair of frontal glands, located anterior to the brain, has been reported by Rieger [10], and is possibly also present in *Lobatocerebrum riegeri* n. sp. (Fig. 7c). As for the posterior frontal glands, their presence could be detected indirectly with CLSM by paired, seemingly empty cavities filled by large cells with elongated nuclei and distinctly tubulinergic ducts. Some of these short ducts opening midventrally did show acetylated alpha-tubulin-IR. However, not all ducts could be traced with certainty to their external ventral openings, since they do not seem to possess the same high density of tubulinergic elements as the ducts of the posterior frontal glands. In the same ventral location of the rostral tip of the animal, TEM showed several tube-like structures with more electron-dense and narrow granules than detected in the tubular glands (Fig. 6f), which are assumed to constitute the secretion of the anterior frontal glands (afg, Fig. 7d).

#### Pharyngeal glands

The major glandular structures of the digestive system are the big, multicellular glands of the pharynx, whose products are secreted in the area of the mouth opening (Fig. 7e). 17–18 elongated ducts (diameter 1.8–3.5 μm, length 70–100 μm, *n* = 3, *r* = 2, *m* = 5, Fig. 7g) of posteriorly located glands surround the mouth opening. They are arranged in a denser pattern in its posterior third , while they are more loosely set anteriorly. The main glandular body can be detected posterior to the mouth opening, on the ventral side of the body dorsal to the ventral nerve cords. It is seen as an elongated, bag-like structure filled with spherical, electron dense granules (1.2–1.7 μm. *n* = 2, *r* = 2, *m* = 5) best detected with FMRFamide-like-IR or TEM (Fig. 7e–g). These glands are not epidermal, and their cell bodies are found inside both the longitudinal musculature and transverse muscular ring complexes of the body wall.

#### Male gonopore glands

Acetylated tubulin-IR was recovered in cells surrounding the dorsal male gonopore. The openings of 16–20 (*n* = 3, *r* = 2–3, *m* = 5) gland cells constituting the complex (Fig. 7h) are connected to the gland bodies via elongated, thin ducts, which are 1.0–1.5 μm in diameter and are all leading to a sunken-in area (14.8–18.3 μm × 6.4–8.2 μm, *n* = 3, *r* = 2–3, *m* = 5, Fig. 7h) around the male gonopore. Approximately half of the cells are densely packed around the anterior end, and the other half around the posterior end, with a small gap between the two portions.

### Reproductive system

*Studied in LM, with acetylated α-tubulin and DAPI staining in CLSM, Fig.* *8*.

In all four adult animals investigated, both male and female reproductive organs or gametes could be found, as well as seminal receptacles to store the mating partner’s sperm.

#### Male gonad

The male gonad is located on the dorsolateral side of the animal, posterior to the third commissure. It is an elongated, thin structure, with the gonopore opening on the dorsal surface of the animal (diameter 1.5–2.7 μm, *n* = 3, *r* = 2–3, *m* = 3, Figs. 7g, 8a). A thin channel (diameter 1.4–1.8 μm, *n* = 3, *r* = 2, m = 5) extends posterior to the pore, with a high amount of the long, thin, fibrous sperm stored in the posterior region (Fig. 8a). Where the sperm is produced is unclear; however, the majority of glands involved in this apparatus are arranged around the gonopore itself, as described above, creating a glandular field (16.2–17.0 μm × 3.5–5.4 μm, *n* = 3, *r* = 2, 3, *m* = 4, Figs. 7a, b, 8a).

#### Female gonad

Up to four eggs, lined up behind each other and increasing in volume posteriorly (Fig. 8b), are the only structures of the female gonad detected with either immunohistochemistry or live observations. The eggs are of irregular shape, reflecting the available space in the body. Although the openings of both seminal receptacles and the male gonad have been found, no obvious opening was detected near the eggs, and they may have to be deposited via rupturing of the epidermis.

#### Seminal receptacles

In the posterior part of the body, the adult animals form one to several seminal receptacles (rs, Fig. 8c, d). These receptacles are thin-walled capsules consisting of few cells without any specific immunoreactivity (Fig. 8c, d). Their diameter is 20–30 μm (*n* = 3, *r* = 1, *m* = 4), and the sperm filaments (spf) can be seen inside, bent and curled up (Fig. 7e). The openings of the receptacles (ors, diameter 0.8–1.7 μm, *n* = 3, *m* = 3) are on the ventrolateral side of the body (Fig. 8c).

### Motility patterns

*Studied in LM, Additional file**1*.

#### Ciliary locomotion

*Lobatocerebrum riegeri* n. sp. is uniformly ciliated along the entire body and moves mainly by a relatively slow, but steady back and forward ciliary gliding rather than muscular action (Additional file 1). Ciliary mode of locomotion is cost-efficient for minute interstitial animals, yet fast reactions to avoid obstacles are dealt with by contractions of the longitudinal (and to a lesser degree transverse musculature ring complexes) body wall muscles.

#### Muscular locomotion

Behavioral observations of several specimens revealed different movement patterns of the rostrum and the remaining body: while the posterior part of the body was often found curled up and attached to the substrate, the anterior part did exploratory movements, including contraction along the longitudinal body axis and sweeping of the rostrum from side to side (Additional file 1). This coincides with the lack of transverse muscular ring complexes and presence of star-shaped muscles in the rostrum. During these contractions of the longitudinal muscles, the anterior part of the body appears more wrinkled, also indicating that an elongation or contraction of the longitudinal muscles in the anterior region is not affecting the trunk and posterior part of the body. With all the longitudinal muscles being continuous along the entire body, the stabilizing and immobilizing of the median body during longitudinal contractions may be accomplished by counteracting contractions of the transverse muscular ring complexes in the trunk and posterior part of the body.

The animals also regularly curl up or fold their posterior body in sinuous curves, which may facilitate anchoring the body among sand grains in the substrate. The trunk may also show minor contractions and winding movements occasionally providing a forward movement in a snake-like pattern (Additional file 1). This most likely is due to a combination of muscular and ciliary locomotion.

The posteriormost end of the body can also be active and flexible (performing contractions and elongations as well as bending movements), though this motility is limited to a small region anterior to the anus (10–30 μm, *n* = 3, *r* = 4, *m* = 5). Occasionally, when the posterior part is curled up or bent, it would act more as an anchor rather than promote forward movement (Additional file 1). *Lobatocerebrum riegeri* n. sp. has never been observed to leave the substrate and swim into the water column.

#### Movements in the digestive system

Although no feeding behavior could be observed, stomach content was moved continuously in both directions, even when the animal was not moving (Additional file 1). This indicates that the weak musculature of the digestive system, maybe together with the body wall musculature, is responsible for movement of the food through the body. The fifth sphincter here probably plays an important role in sealing the digestive tract and prohibiting food getting expelled through the pharynx and mouth opening again, since no movement of food could be observed in the pharynx anterior to this muscular constriction.

## Taxonomy

Phylum Annelida Lamarck, 1809

Family Lobatocerebridae Rieger, 1980

Genus *Lobatocerebrum* Rieger, 1980

Species *Lobatocerebrum riegeri* n. sp.

(Figs. 1, 2, 3, 4, 5, 6, 7, 8, 9, Tables 1, 2, 3, 4, Additional file 1)

**Table 1:** Measurements of the specimen of *Lobatocerebrum riegeri* n. sp. investigated in this study and distances of specific structures and organs to the anterior end of the body

	*End of measurement from the anterior tip*	*Lobatocerebrum riegeri II (CLSM, holotype)*	*L. riegeri III (CLSM, paratype)*	*L. riegeri IV (CLSM, paratype)*	*L. riegeri I (juvenile, CLSM, paratype)*	*L. riegeri V* alive *(LM)*
Total Length [μm]		1571,9	1078	1606	478	1646,6
Total Width [μm]		40	79	55	66,5	51,4
Position of neuropile		247	196	204	132	250
Position of the brain						
	Middle of the brain	246	221	204	137,7	266
	Most anterior part	177	179	159	108	215
	Most posterior part	296	251	248	162	304
Position of the mouth						
	Middle of the mouth	322	374	344	182,3	330
	Most anterior part	305	368	293	170	310
	Most posterior part	343	403	375	203	350
Position of the male gonopore		593	476	557		596
Position of the testis						
	Middle of the testis	758	576,5	712		776
	Most anterior part	725	556,5	691		741
	Most posterior part	787	596,5	732		811
Position of the ovary						
	Middle of the ovary	1107	702,5	1053		1150
	Most anterior part	953	597,8	1036		995
	Most posterior part	1248	769,5	1070		1304
Position of the seminal receptacles						
	Middle of the receptacles	1428	960,5	1350		1510
	Most anterior part	1424,9	944,5	1340		1501
	Most posterior part	1432,9	970	1360		1519

The measurements were taken from both live (*n* = 1) and fixed and mounted (*n* = 5) specimens, including one juvenile, as indicated. In the latter, neither the male nor the female gonad could be detected in transmitted light or CLSM-images. Measurements are taken in μm (in case of body length and width) and as μm from the anterior end of the respective animal to a specific point as indicated in the first and second column

**Table 2:** Comparisons of measurements and distances of specific structures and organs to the anterior tip of different species of Lobatocerebridae

	*Lobatocerebrum psammicola* live	*L. psammicola* fixed	*Lobatocerebrum* sp. 1	*Lobatocerebrum* sp. 2	*Lobatocerebrum riegeri*	*L. riegeri* conclusions/remarks
Total length [mm]	3.0	2.0–2.2	1.1	1.7	1.57 (1.08–1.6 [0.48])	*L. riegeri* is shorter than *L. psammicola* and the other reported specimens
Total width [mm]	0.11	0.07–0.08	0.06	0.06	0.04 (0.04–0.06 [0.07])	*L. riegeri* is thinner than the other species and reported specimens, though not relative to the body length
Relative width	0.036	0.035–0.036	0.055	0.035	0.025 (0.025–0.038 [0.15])	*L. riegeri* is thinner than the other species and reported specimens, though not relative to the body length
Position of the neuropile [1–100U]	9	7–12	14	12	18.18 (8.22–18.18 [27.61])	→displaced more posteriorly in *L. riegeri* than in *L. psammicola* and the other reported specimens
Position of the brain [1–100U]	9	7–12	14	12	15.65 (12.7–20.5 [28.8])	
Position of the mouth [1–100U]	14	10–17	20	20	20.48 (20.48–34.69 [38.14])	→displaced more posteriorly in *L. riegeri* than in *L. psammicola*, but in the same range as the other reported species
Position of the male gonopore [1–100U]	38	30–36	No measurements provided	31	37.72 (34.68–44.16)	→ range outside *L.* sp. 2, but similar to *L. psammicola*
Position of the testis [1–100U]	47–57	46–56	No measurements provided	35–43	48.21 (44.33–53.48)	→ posterior to *L.* sp.2, but with the broad range similar to *L. psammicola*
Position of the ovary [1–100U]	58–63		No measurements provided	48–79	70.42 (65.17–70.42)	→ too broad ranged to be diagnostic
Position of the seminal receptacles [1–100U]	90,5	87–89	No measurements provided	88	90.84 (84.06–90.84)	→ too broad ranged to be diagnostic

The measurements of *Lobatocerebrum psammicola*, *L.* sp. 1 and *L.* sp. 2 were taken from [8]. *L. riegeri* n. sp*.* (this study) was obtained from this study and translated in the units used by [8] (in 1–100U for the entire body length). For *L. riegeri* n. sp., all measurements are taken from fixed and mounted specimens in the following order: holotype [range of all adult specimens (juvenile)]. *L. riegeri* n. sp. specimen III was excluded from the range given for body length and width, since it was compressed to a high degree, but was considered for the relative measurements

**Table 3:** Compilation of features of the nervous system in representatives of different spiralian groups with previously proposed relationship to *Lobatocerebrum riegeri* n. sp

		ANNELIDA			MOLLUSCA			NEMERTEA			GNATHOSTOMULIDA			PLATYHELMINTHES	XENACOELOMORPHA
		LOBATOCERBRIDAE	SIPUNCULA	ORBINIIDAE	SOLENOGASTRES	CAUDOFOVEATA	GASTROPODA	PALAEONEMERTEA		ANOPLA	BURSOVAGINOIDEA		FILOSPERMOIDEA	CATENULIDA	NEMERTODERMATIDA
		*Lobatocerebrum riegeri* n. sp.	*Phascoliun strombus*	*Scoloplos armiger*	*Dorymenia sarsii*	*Chaeoderma japonicum*	*Helminthope psammibionta*	*Cephalothrix linearis*	*Procephalo-thrix linearis*	*Lineus viridens*	*Gnathostomula peregrina*	*Rastrognathia macrostoma*	*Pterognathia meixneri*	*Stenostomum leucops*	*Nemertoderma westbaldi*
	Location of the ventral nerve cords	Intraepithelial	Intraepithelial/subepidermal	Intraepithelial	Subepidermal	Subepidermal	Subepidermal	Subepidermal	Subepidermal	Subepidermal	Intraepithelial	Intraepithelial	Intraepithelial	? (mainly subepidermal)	Intraepithelia to subepidermal
BRAIN	Lobular structure	+	?	+	-	+	+	+	+	+	-	? (−)	-	-	-
	Central neuropile	+	+	+	+	+	+	+	+	+	+	? (+)	+	+	- (only commissures formed)
	Number of brain commissures	4	2	4	1	1	1	>2	>2	>2	1	? (1)	1	1	2 rings (dorsally connected)
NERVE CORDS OF THE CENTRAL NERVOUS SYSTEM	Number of posterior longitudinal nerve cords	1 pair + 1 median cord	1 pair	1 pair	2 pairs (+1 median cord)	2 pairs	2 pairs	1 pair	1 pair + 1 dorsal median + 1 ventral median cord	1 pair	1 pair	1 pair	1 pair	1 pair	- (thin fibres, but no cords)
	Median posterior nerve cord	+	-	-	+	-	-	-	+	-	+ (just a short piece)	? (−)	-	-	-
	Number of rostral longitudinal nerve cords	2–9	0	0	-	-	2 pairs	?	4	Approx. 8 pairs	-	?	>3 paired and 2 unpaired	-	-
GANGLIA AND COMMISSURES ALONG THE VENTRAL NERVE CORD	Total number of ganglia	2 pairs	>2	>2	>2	>2	>2	?	?	1 pair	1 pair	? (1 pair)	1 pair	?	?
	Nonganglionated posterior commissures	>2	?	>2	>2	>2	>2	?	1	?	1	? (1)	1 (2)	?	-
	Presence of a subpharyngeal ganglion	+	+	+	+	+	+	+	+	+	+	? (+)	+	+	-
PERIPHERAL NERVOUS SYSTEM		Grid of distinct longitudinal and circular nerves	Nerve plexus	Grid of pairwise arranged longitudinal and several circular nerves per segment	?	?	?	?	Intraepidermal plexus around the rhynchocoel	Subepidermal plexus, commissural plexus, stomatogastric plexus, proboscidial plexus	5 longitudinal nerves	6 longitudinal nerves	3 dorsal longitudinal nerves	?	?
References		This study	[57, 73, 74]	[75, 76]	[40, 41]	[40]	[43]	[42, 48]	[42]	[77]	[78]	[17, 79]	[17]	[46, 80]	[81, 82]

Details of the brain, the ventral nervous system, the stomatogastric nervous system and the peripheral nervous system are given in an attempt to reveal common features or possible apomorphies in Lobatocerebridae. Presence of a character is labeled with +, absence with -, numbers and additional informations are given wherever possible. “?” indicates the lack of information in the references mentioned, while reinvestigations from this study (in the case of *L. riegeri* n. sp.) and assumptions based on additional references are included by putting the assessment in brackets (+) or (−). Only species with previously [8–12] or recently [7] suggested relationship to Lobatocerebridae were considered. Insufficient information in one species was supplemented with closely related species, based on the literature acknowledged in the reference-row

**Table 4:** Compilation of features of the nervous system in representatives of different annelid groups and *Lobatocerebrum riegeri* n. sp

		ANNELIDA												
		PREVIOUS “PROBLEMATICA”, now ANNELIDA												
		LOBATOCEREBRIDAE		DIURODRILIDAE	?	SIPUNCULA		DINOPHILIDAE	PROTODRILIDAE	PSAMMODRILIDAE	NEREIDIDAE	CAPITELLIDAE	SERPULIDAE	
		*Lobatocerebrum riegeri* n. sp.	*Lobatocerebrum psammicola*	*Diurodrilus* sp.	*Jennaria pulchra*	*Phascolion strombus*	*Siphonosoma australe*	*Dinophilus gyrociliatus*	*Protodrilus* sp.	*Psammodrilus fauveli*	*Platynereis* sp.	*Capitella sp.*	*Pomatoceros lamarckii*	*Spirorbis* cf. *spirorbis*
	Location of the ventral nerve cords	Intraepithelial	Intraepithelial	Intraepithelial	Intraepithelial	Intraepithelial	Intraepithelial	Intraepithelial	Intraepithelial	Intraepithelial	Intraepithelial	Intraepithelial	Intraepithelial	Intraepithelial
BRAIN	Lobular structure	+	+	?	+	?	?	-	-	+	+	+	-	+
	Central neuropile	+	+	+	+	+	+	+	+	+	+	+	+	+
	Number of brain commissures	4	?	4	?	2	1	2	4	4	4	4	4	4
	Dorsal root (dorsal/ventral commissure)	+ (+/+)	?	+ (+/+)	?	?	- (Not differentiated in this species)	?	+ (+/+)	+ (+/+)	+ (+/+)	+ (+/+)	+ (+/+)	+ (+/+)
	Ventral root (dorsal/ventral commissure)	+ (+/+, individual fibres spread out)	?	+ (+/+)	?	?	- (Not differentiated in this species)	?	+ (+/+)	+ (+/+)	+ (+/+)	+ (+/+)	+ (+/+)	+ (+/+)
NERVE CORDS OF THE CENTRAL NERVOUS SYSTEM	Number of posterior longitudinal nerve cords	1 pair + 1 median cord	1 pair (+1 median cord?)	2 pairs	1 pair	1 pair	1 pair (fused during development)	3 pairs + median cord	1 pair	1 pair	1 pair	1 pair	1 pair	1 pair
	Median posterior nerve cord	+	?	-	-	-	-	+	-	-	-	+	+	-
	Number of rostral longitudinal nerve cords	2 ventrolateral + < 7 additional, smaller ones	2 (?)	>2	?	0	0	0	0 (but innervation of tentacles)	0	0	0	0 (but innervation of tentacles)	0 (but innervation of tentacles)
GANGLIA AND COMMISSURES ALONG THE VENTRAL NERVE CORD	Total number of ganglia	2 pairs	2 pairs	1 (fused pair)	?	>2	>2 (during development)	>2	>2	>2	>2	>2	>2	1 (in larvae)
	Nonganglionated posterior commissures	>2	2	>2	1	?	>2 (during development)	>2	>2	>2	>2	>2	>2	1 (in larvae)
	Presence of a subpharyngeal ganglion	+	+	+	?	+	+ (during development)	+	+	+	+	+	+	+
STOMATOGASTRIC NERVOUS SYSTEM	Stomatogastric nervous system	+ (ring around the pharynx)	+ (ring around the pharynx)	+ (ring around the esophagus)	+ (nerve cells in the pharyngeal epithelium)	+ (ring around the esophagus	+ (ring around the esophagus, during development)	+ (ring around the pharynx)	+ (ring around the esophaus)	+ (ring around the esophagus, also tracing the esophagus)	+ (ring around the esophagus)	+ (ring around the esophagus)	+ (fibre along the gut, ring around the esophagus)	-
	Origin of the stomatogastric nervous system	Postpharyngeal ganglion	Postpharyngeal ganglion	prebuccal ganglion	?	brain (?)	brain (?)	Brain (dorso-posterior neuropile)	Brain	Buccal ganglion	Brain	Brain	Brain	-
PERIPHERAL NERVOUS SYSTEM		Grid of distinct longitudinal and circular nerves	?	1 pair of longitudinal nerves, several branches for innervating organs	Some nerves around the pharynx and gut, otherwise not present or not described	Nerve plexus	?	Regular grid of longitudinal and circular nerves, nerve plexus dorsal to the ventral nervous system	?	?	Grid of distinct longitudinal and circular nerves	Grid of distinct longitudinal and circular nerves	Circular nerves in some segments	Grid of distinct longitudinal and circular nerves
References		This study	[8–11]	[61]	[9, 83]	[57, 73]	[58]	[14, 84, 85]	[55, 86]	[87, 88]	[70, 89, 90]	[91]	[92]	[92]

Details of the brain, the ventral nervous system, the stomatogastric nervous system and the peripheral nervous system are given in an attempt to reveal common features or possible apomorphies in Lobatocerebridae. Presence of a character is labeled with +, absence with -, numbers and additional informations are given wherever possible. ? indicates the lack of information in the references mentioned, while reinvestigations from this study (in the case of *L. riegeri* n. sp.) and assumptions based on additional references are included by putting the assessment in brackets (+) or (−). Insufficient information in one species was supplemented with closely related species, based on the literature acknowledged in the reference-row

*Lobatocerebrum* sp. 2 in [8–11], registered in ZooBank (E3DCE97A-7F7A-4799-827A-DF2EA41AE1A5).

### Diagnosis

Entirely ciliated *Lobatocerebrum*, unsegmented, hyaline body with glandular epidermis (unicellular, kidney-shaped glands with transparent-green content), 1.08–1.6 mm in length and 0.04–0.06 mm in diameter. Large, lobular brain, with central neuropil displaced 8.22–18.18 U posterior of anterior body edge (relative to total body length). Ventral mouth opening, positioned posterior of the brain, 20.48–34.69U from anterior edge (relative to total body length). Dorsal opening of male gonopore positioned 10-14U posterior to the neuropil (relative to total body length).

### Type material

Holotype: one 1.57 mm long mature hermaphrodite (testis, ovary with eggs and seminal receptacles present) (ZMUC-POL-2384), beach in front of the Interuniversitary Institute for Marine Sciences (IUI) northwest of Eilat, Israel (N 29° 30.211’ E 34° 55.068), 9 meters deep, coral sand, collected by the authors 20.02.2014. Paratypes: Two mature and one juvenile specimens (section series, ZMUC-POL-2385, ZMUC-POL-2386, ZMUC-POL-2387), same locality as for holotype, (sampled on 14.02.2014, 16.02.2014 and 18.02.2014); one mature specimen collected by Mike Crezée (section series, ZMUC-POL-2388).

### Etymology

The species is named in memory of Reinhard M. Rieger, who discovered and described the first representative of Lobatocerebridae.

### Description

Measurements of holotype are given in the text, ranges of all types are given in parentheses; juvenile is not included)

*Lobatocerebrum riegeri* has an elongated, cylindrical, entirely ciliated body, which appears slightly greenish due to the glandular epidermis (Fig. 9a). The total body length is 1.57 mm (varies between 1.08 and 1.6 mm in adults), the body width is 0,04 mm (0.04–0.06 mm, Tables 1, 2). The rostrum is 305 μm (293–368 μm, Fig. 9b); the uniform trunk extends for an additional 1266 μm (710–1336 μm, Table 1). The brain is located dorsally in the rostrum 246 μm (204–266 μm) from the anterior tip, extends for 119 μm (30–44 μm) posteriorly and has an oval, but lobular appearance (two frontal and four posterior lobes embracing the central neuropil visible with LM, Fig. 9c, Tables 1, 2). The mouth opening is 322 μm (330–374 μm) from the anterior tip; extends for 21 (20–31 μm, Fig. 9d, Tables 1, 2) and the pharynx is heavily ciliated and supplied with several glands. The transitions from the fore- to the mid-gut 480 μm (450–580 μm) from the anterior tip and from the mid- to the hindgut 820 μm from the anterior tip (800–1300 μm) are marked by a decrease in diameter, sphincter muscles and change in ciliation pattern (strong in fore- and hind-gut, weaker in mid-gut). No protonephridia were detected with the techniques applied (adults and juvenile). The male gonopore 593 μm (476–596 μm) from the anterior tip and associated gland cells as well as one testis 758 μm (576–758 μm, Fig. 9a, Tables 1, 2) from the anterior tip are all located dorsally. In mature specimens, big, slightly oval but irregular-shaped eggs can be found in the posterior region of the body 1107 μm (702–1107 μm, Tables 1, 2) from the anterior tip). Seminal receptacles, if present (one to three found in the specimens investigated), can be found in the posterior region of the body 1428 μm (960–1428 μm, Table 1) from the anterior tip, opening laterally. The anus opens dorsally 1500 μm (1000–1500 μm, Fig. 9e) from the anterior tip.

### Remarks

*Lobatocerebrum riegeri* is smaller (1.08–1.6 mm in adults compared to 2.0–3.0 mm in adults of *L. psammicola*) and thinner (0.04–0.06 mm in adults compared to 0.07–0.11 mm in *L. psammicola*) than the related species [8]. The brain is displaced more posterior (8.22–18.18 U (distance from anterior end to central neuropil relative to total body length) in adults compared to 7-12U in *L. psammicola*) and the mouth opening is displaced further posterior in the body than in the previously described species (12.7–20.5U in adults compared to 10-17U in *L. psammicola*). Further distinguishing *Lobatocerebrum riegeri* from its previously described relative is the fact that it has a different secrete in the unicellular adhesive glands (linearly arranged globular inclusions in the granules in the adhesive glands in *L. riegeri* as compared to linear, rod-shaped inclusions in *L. psammicola*). Additionally, the two localities the different species have been found in (North Carolina, USA for *L. psammicola* and Eilat, Israel for *L. riegeri*) are far apart from each other and therefore the presence of two species seems to be probable. Further studies also involving molecular data are needed to further support this hypothesis, but are unfortunately not available now.

## Discussion

### Function and origin of the unique muscular ring complex

The characteristic annelid (and spiralian) muscular arrangement consists of an external circular and internal longitudinal muscle layer [22, 38]. However, the pattern in Lobatocerebridae differs in having externally positioned longitudinal muscles sunken into the epidermis, and within those inner transverse muscles previously mistakenly interpreted as continuous circular muscles [8]. However, each of these ring complexes resembles a discontinuous muscular network, composed by transverse muscle fragments, which together form serially repeated, discontinuous muscular ring complexes interconnecting the longitudinal muscles. Peristaltic body movements normally caused by contraction of circular muscles where never observed in *Lobatocerebrum riegeri*; however, the transverse fragments neither seemed to operate independently, but most likely aid to stabilizing the body wall during contraction of the longitudinal fibers. The lack of ring complex muscles in the rostrum on the other hand seems to allow for the high flexibility of the long rostral area in *L. riegeri* (Fig. 1b, e–g, Additional file 1). A flexibility which otherwise would have been prevented due to their different interconnecting composition compared to regular spiralian circular muscles, located external of the longitudinal muscles, even along the long rostrum of meiofaunal animals such as a the filospermoid Gnathostomulida [17] and catenulid Platyhelminthes ([39], Table 3). Since a similar muscular solution to both granting flexibility of the rostrum and stabilizing the trunk is not found in other annelids (or sipunculids), the muscular ring complex is considered a unique apomorphy of Lobatocerebridae.

### The paradox of a complex brain in a simple animal

Lobular or compartmentalized, ganglionated brains are commonly found in macroscopic representatives of Spiralia and other metazoan groups (e.g. [18, 40, 41]), but interstitial animals generally do not show such a complex architecture (e.g. [16, 29], Tables 3, 4). However, some interstitial species of nemerteans [42], molluscs (especially in several wormlike gastropods such as *Helminthope* [43], *Rhodope* [44], and *Pseudovermis* [45]) and catenulids [46] also show some compartmentalization of the brain having, for example, visual and olfactory centers (Table 3). Another representative with a ganglionated brain is the enigmatic interstitial “worm” *Jennaria pulchra* (Figure 3a in [9])*,* which is described as representing many plesiomorphies of the trochozoan body plan [47] and possibly being an annelid [9]. Different compartments or lobes of the brain are normally related to processing of different sensory stimuli, yet all conspicuous sensory organs such as eyes, sensory appendages or olfactory nuchal organs are lacking in Lobatocerebridae. Moreover, the indistinct gut content and simple alimentary tract and behavior indicates that *Lobatocerebrum sp.* is an unselective deposit feeder. Though no sensory structures are found adjacent to, or directly connected to specific regions in the brain, it is still striking how the anterior rostrum is strongly innervated with nerves connected to various parts of the brain. Hence, though unlikely, the glandular secretion or the stimuli of the scattered sensory cells may in fact be processed in a much more organized manner and their signaling complexity exceed our expectations. Nonetheless, the complex lobular architecture of the brain in *L. riegeri* seems a functional paradox.

### Systematic importance of longitudinal nerve configuration

Annelid central nervous systems vary in numbers of main longitudinal nerves, from one ventro-median cord to seven or more ventral nerves (Table 4, [21]). Based upon developmental studies and a broad comparison across Annelida, five ventral cords have been proposed as the ancestral pattern [15], yet this proposed character evolution was never traced upon a phylogenetic tree. The pattern of five nerves is made up of one pair of ventral, one pair of lateroventral and one median cord. The latter is revealed during neurogenesis in several annelids, and has been found in most interstitial annelids, possibly being an annelid apomorphy. However, it is only found elsewhere in Spiralia in a few exceptional cases (and with somewhat different configuration) (Solenogastres [40], some Nemertea [48], Table 3). According to a parsimonious tracing on the latest Spiralian tree [7] one pair of widely separated ventral cords would be the plesiomorphic state of Spiralia (Table 3 and references therein). Likewise, the basi- or intraepidermal position of the nervous system has also been regarded a plesiomorphic trait in Spiralia [49] as well as in Annelida such as now exemplified by the early branching annelid lineage Oweniidae [50–52] opposed to the derived subepidermal position found in many crown group annelids [53, 54]. However, intraepidermal nerve cords have also been found in Siboglinidae (Worsaae K, Rimskaya-Korsakova, NN, Rouse, GW: Neural reconstruction of bone-eating *Osedax* spp. (Annelida) and evolution of the siboglinid nervous system, submitted) as well as several interstitial annelids [19, 25, 54], showing considerable variance throughout evolution. The intraepidermal position of the paired ventral cords of *Lobatocerebrum* may hereby not be phylogenetically informative, whereas its additional median cord may be an annelid apomorphy. The two widely separated main nerve cords do not resemble a “typical” annelid pattern, but also do not dispute such a relationship, since such a pattern is also found in several other interstitial annelids such as Dinophilidae [15], Protodrilidae [29, 55], and Nerillidae [56].

*Lobatocerebrum* also possess two prominent and several additional long rostral nerves extending from the posteriorly displaced brain to the tip of the animal ([8], this study). This pattern is not found in any other annelids, which normally have the brain located anteriorly [28]. A similar pattern is found in a few examples of distantly related interstitial spiralians among Mollusca, Nemertea, Gnathostomulida, Catenulida (for details see Table 3) but has most likely arisen by convergence.

### Lobatocerebrum – an unsegmented annelid?

The ventral nervous system in annelids most commonly consists of longitudinal nerve cords linked by ganglionated, serially arranged commissures, correlated with other serially repeated structures to form segments [49]. However, a clear outer segmentation as well as regularly distributed segmental paired ganglia are lacking in several groups recently assigned to annelids such as Diurodrilidae, Sipuncula, Echiura Siboglinidae and now also demonstrated for *Lobatocerebrum riegeri*. A similar layout to that of *L. riegeri* only having two pairs of subpharyngeal ganglia is also found in other spiralian groups (e.g. Gnathostomulida, Catenulida, for more details see Table 3), although the posterior commissures found in *L. riegeri* (ganglionated and non-ganglionated) are often not described or irregularly distributed (Table 3). Besides the low number of ganglia, there is no correlation of the commissural distribution with that of the few observed nephridia in *L. psammicola* [8, 9] nor with any other organ system in *L. riegeri*, which means that *Lobatocerebrum* cannot be regarded as segmented at present. This emphasizes, however, that more detailed studies of the developmental pattern in Lobatocerebridae are needed to check for signs of segmentation during ontogeny as found in Echiura and partly in Sipuncula [57, 59].

### A grid-like peripheral nervous system supporting a ventralized central nervous system may be a Spiralian plesiomorphy

The peripheral nervous system is, especially in spiralians with a ventralized central nervous system, supposed to provide sufficient support and innervation for (sensory) organs in the periphery of the body [49]. Especially sensory cilia and glands are often abundantly distributed in the epidermis of interstitial animals far from the ventral nerve cords and the brain, as can be demonstrated in nearly all spiralian groups [60]. In annelids, the peripheral nervous system is often formed as an irregular grid constituted by longitudinal, oblique and circular nerves [21], relatively similar to those present in *L. riegeri*, though the pattern here appeared more regular and with the longitudinal nerves projecting directly from the neuropil rather than from the nerve cords. Moreover, this is the general pattern for several spiralians, so it cannot be viewed as a diagnostic trait for annelids (see Tables 3 and 4 for details). Supplementing or even replacing this grid, nerve plexi are found around specific organs, most often adjacent to the (male) reproductive organs or the mouth opening in nearly all groups considered for this comparison (see Tables 3 and 4 for details). However, since the grid is generally built from single (or few) fibres, the record of peripheral nervous system architecture especially among interstitial animals is rather incomplete.

### Function and origin of the long-necked frontal glands

The frontal glands in *Lobatocerebrum* are among the diagnostic features of this group; the elongated ducts of the prominent glands can neither be found in other annelid groups (with the exception of Diurodrilidae [61]) nor in the majority of other spiralian groups. However, supposedly similar structures are present in catenulid Platyhelminthes (personal observation) and probably also in a few exceptional nemerteans and gnathostomulids (W. Sterrer, personal observation). The function of these glands is still unclear, though two options seem most likely: i) the secretion of these glands is used to produce a mucus layer to facilitate ciliary gliding; ii) the secretion is used to bind substances (e.g. pheromones or other chemical compounds) from the environment and thereby enhance the animal’s ability to sense the environment and possibly even follow a chemical lead. However, though olfactory organs have been described for many invertebrates [62, 63], with annelids generally having ciliated nuchal organs [64–66], those are rarely glandular or resembling the structure of the frontal glands, why this hypotheses clearly needs further testing.

### Origin of meiofaunal characteristics of Lobatocerebridae

Lobatocerebridae has been proposed to originate from a macroscopic, presumably annelid (or annelid-like) ancestor by progenesis (somatic arrest during larval or juvenile development due to early maturation [67]) [8, 9, 11]. This idea was based on its acoelomic condition and the presence of characters also present in annelid or spiralian larvae, such as complete ciliation, an intraepithelial nervous system, protonephridia and a rather simple formation of both musculature and ventral nervous system [8, 9, 11]. No single extant macrofaunal lineage possesses juveniles resembling adult Lobatocerebridae; however, the noted features are also common in other meiofaunal representatives of annelids, molluscs, nemerteans and platyhelminths (see Tables 3 and 4, and references herein for details), where progenesis is often seen as the most plausible pathway along which these interstitial animals have derived from a macroscopic ancestor [67]. Conversely, most of these features are also present in the early branching meiofaunal spiralian lineages (Gnathifera, Platyhelminthes, Gastrotricha) and were, according to the latest Spiralian topology, proposed to resemble spiralian plesiomorphies [7]. So when these traits are found in adult meiofauna they may not necessarily reflect an ancestry from a larval or juvenile stage, but could instead represent plesiomorphic states – or as a third alternative, gradual adaptations (reversals) to the constraints of the space-restricted interstitial environment [11, 16, 30, 31].

Meiofaunal spiralians generally have few nerve cords spaced far apart (rather than midventrally fused/condensed), and possess a body wall musculature spread out as a regular grid (rather than having the longitudinal muscles organized into four or fewer bundles, see Tables 3 and 4 and references therein for details). Besides this pattern possibly being the ancestral spiralian condition, there may exist ‘universal constraints’ on the functionally optimal neuromuscular design when being of microscopic size and with limited cell number, and given the evolutionary toolbox within Spiralia. Hence, the organization of the neuromuscular system may be more directly dependent on e.g., size, ciliary pattern or acoelomatic condition (e.g. as for the mesodermal blood vascular system and protonephridia [11, 68, 69]) in a way we haven’t calculated for. Alternatively (or in addition), the condensation of muscles and nerves into bundles is a pattern often realized during development of annelids and certain spiralians (e.g., [70–72]), and although there is currently no way of testing this statement, the lack of condensation in *Lobatocerebrum* may also be seen as an evolutionary arrest in somatic development (at least of these specific somata) and hereby as a sign of paedomorphic origin rather than gradual adaptation. However, in the recent phylogenomic study placing *Lobatocerebrum* within Annelida [7], the exact position is not well supported, which is why its descend from either macrofaunal or a meiofaunal ancestor cannot be traced with conviction.

## Conclusion

Although *Lobatocerebrum* was shown to be an annelid in a recent phylogeny [7], previous studies also suggested similarities to other spiralian groups such as Platyhelminthes, Nemertea, Mollusca and Gnathostomulida [8, 10, 11]. Conducting a detailed study of *Lobatocerebrum riegeri* with several complementary microscopical techniques revealed details of the musculature, the nervous system and the glandular system and allowed for a detailed description of *Lobatocerebrum riegeri* next to the previously described *L. psammicola*. Yet, *L. riegeri* is very similar to *L. psammicola*, both representing conservative spiralian patterns and a combination of traits diagnosing it as an annelid. Most features of the neuromuscular system revealed in *L. riegeri* by CLSM and TEM are not in themselves diagnostic to annelids and can either likewise be found in other groups or be unique for Lobatocerebridae. While these features on their own cannot reveal significant information about relationships within and between the spiralian groups, the combination of traits such as a nervous system with a complex brain with several commissures, a prominent median nerve cord and several ganglionated commissures, as well as a glandular, multiciliated epidermis and gliointerstitial system [10] together support an affinity to Annelida.

It is not possible to depict neither from the phylogenetic position nor morphological traits whether Lobatocerebridae originated through paedomorphosis or gradual miniaturization from a macrofaunal ancestor as an adaptation to the interstitial environment - or may even have retained plesiomorphic traits. Nonetheless, the lack of specific resemblance to any juvenile annelid relatives indicates a much more complex evolutionary history than what can be explained by a one-step progenetic evolutionary process. Further studies on the development of organ systems such as the musculature and the nervous system may prove useful for accessing the origin of Lobatocerebridae. Nonetheless, this study demonstrates that with Lobatocerebridae being annelids [7], Annelida displays an extreme evolutionary plasticity of the neuromuscular system, which is otherwise regarded as highly conservative throughout metazoan evolution.

## Methods

### Sampling

Specimens used for this study were collected in Eilat, Israel, from sand collected from a small (0.5x0.5 m) sand patch between coral blocks at 8.5–9 m depth approximately 100 m southwest of the main pier of the Interuniversitary Institute for Marine Sciences (IUI, N 29° 30.211’ E 34° 55.068). Animals were extracted and anesthetized using an isotonic magnesium chloride solution: The upper 2–5 cm of sampled sand was mixed with this solution, and the water with floating particles and anesthetized animals decanted through 63 μm meshes with seawater. Revitalized animals were sorted from the petri dish using dissecting compound microscopes. A total of nine specimens was found, examined and afterwards fixed for the techniques described below as well as for molecular analysis.

### Behavioral studies

Animals were observed with a dissecting scope in a petri dish prior to being transferred to an object slide in seawater under cover for examination and imaging in a compound microscope with a mounted camera or a video recorder. For later relaxation, a weak MgCl_2_-solution was added to the slide. Movies were later analyzed in relation to the morphological studies and interpretation.

### Histology, light microscopy (LM) and transmission electron microscopy (TEM)

Specimens were carefully anesthetized with isotonic magnesium chloride and afterwards fixed with 2 % glutaraldehyde in 0.1 M osmolarity-adjusted cacodylate buffer over night at room temperature (RT) and afterwards rinsed and stored in 0.1 M cacodylate buffer. The animals were postfixed in 2 % OsO_4_ in 0.05 M K_3_FeCN_6_-solution for 1 h and before embedding in Araldite Epon-812 using standard protocol and polymerization for 20–24 h at 50 °C.

For TEM-analysis, the block was trimmed to the object and sectioned into 40 nm sections using a Leica EM UC7 ultratome (LEICA MICROSYSTEMS, Wetzlar, Germany). Ultrathin section were mounted on Formvar-coated 2x1mm slot grids, contrasted with 2 % uranyle acetate- and 4 % lead citrate-solution and examined using a JEOL JEM 1010-Transmission Electron Microscope (TEM, JEOL Ltd., Tokyo, Japan) in combination with a digital GATAN OneView camera (GATAN, INC., Pleasanton, CA, United States). The fixation and preparation caused artifacts such as the slight separation of the epidermis from the internal organs of the animal.

### Immunohistochemistry and CLSM

Specimens were carefully anesthetized with isotonic magnesium chloride and afterwards fixed in 3.7 % paraformaldehyde in phosphate buffered saline (PBS) for 1 to 2 h at RT, followed by several rinses in PBS and storage in PBS with 0.05 % NaN_3_. For the investigation of muscular, nervous, glandular and ciliary system quadruple stainings were applied, including F-actin staining (Alexa Fluor 488-labelled phalloidin, INVITROGEN, Carlsbad, USA), DNA-staining (405 nm fluorescent DAPI) and immunostaining (monoclonal mouse anti-acetylated α-tubulin (SIGMA T6793, St. Louis, USA), polyclonal mouse anti-synapsin 1 (3C11 (anti SYNORF1, DEVELOPMENTAL STUDIES HYBRIDOMA BANK, Iowa, USA) and anti-tyrosinated tubulin (SIGMA T9028), polyclonal rabbit anti-serotonin (5-HT, SIGMA S5545) and anti-FMRFamide (IMMUNOSTAR 20091, Hudson, USA)). Prior to adding the primary antibody-mix, the samples were pre-incubated with 0.1 % PBT (PBS + 0.1 % Triton-X, 0.05 % NaN3, 0.25 % BSA, and 10 % sucrose) for 2 h. Afterwards, samples were incubated for up to 24 h at RT in the primary antibodies mixed 1:1 (in a final 1:200 concentration (or 1:50 for anti-synapsin 1)). Subsequently, specimens were rinsed in 0.1 % PBT three to six times and incubated with the appropriate secondary antibodies conjugated with fluorochromes (also mixed 1:1 in a final concentration of 1:200; goat anti-mouse labeled with CY5 (JACKSON IMMUNO-RESEARCH, West Grove, USA, 115-175-062), goat anti-rabbit labeled with TRITC (SIGMA T5268)) for up to 24 h at RT. This step was followed by several rinses in 0.1 % PBT and post-incubation for 60 min in Alexa Fluor 488-labeled phalloidin (0.33 M in 0.1 % PBT). Thereafter, specimens were rinsed in PBS (without NaN3) and mounted in Fluoromount-G with DAPI (SOUTHERN BIOTECHNOLOGY ASSOCIATES, Inc., Alabama, USA) or Vectashield with DAPI (VECTOR LABORATORIES, Burlingame, USA).

The mounted specimen were scanned using a Olympus Fluoview FV-1000 confocal laser scanning microscope (of K. Worsaae, University of Copenhagen, Denmark), with the acquired z-stacks of scans being either projected into 2D-images or analyzed three-dimensionally using IMARIS 7.1 (BITPLANE SCIENTIFIC SOFTWARE, Zürich, Switzerland). This software package was also used to conduct the measurements presented in the following text (*n* = number of specimens analyzed; *r* = body region (1 - from the anterior tip to the mouth opening, 2 - from the mouth opening to the male gonopore, 3 - from the male gonopore to the ovary, 4 - from the ovary to the posterior tip of the animal); *m* = number of measurements per region).

### Measurements

All measurements on live animals were taken in Adobe Photoshop after the images were acquired using a standardized scale bar, as was the procedure for measurements taken from TEM-pictures. Measurements from CLSM-image stacks were conducted in Imaris 7.1 using the *Measurement*-tool in *Section*-mode. For comparison with the measurements in Rieger [8], distances from the rostral tip to specific organ systems as well as body width and length were calculated in units (U), the entire body length being 100U.

### Photoshop and Illustrator

Contrast and brightness of all two-dimensional projections of confocal data and pictures of TEM-sections were adjusted in Adobe Photoshop CC 2015. Schematic drawing as well as plate-assembly was performed in Adobe Illustrator CC 2015.

## Additional file

Additional file 1:
**Motility pattern and details of the adult** ***Lobatocerebrum riegeri*** **n. sp.** This movie shows combined clips of alive *Lobatoerebrum riegeri* n. sp. indicating both morphological specificities such as the pharynx and brain and motility patterns. (MP4 116178 kb)

## Abbreviations

ac: anterior cilia
adnc: anterio-dorsal nerve cord
adlnc: anterior dorso-lateral nerve cord
afg: anterior frontal gland
ag: adhesive granule
amf: anterior point of muscle fusion
an: anus
anc: anterior nerve cord
avnc: anterio-ventral nerve cord
avlnc: anterior ventro-lateral nerve cord
bl: basal lamina
br: brain
brc: brain cell
bsm: brain supporting muscle
c: cilium
c1-4: commissures 1–4
cg: ciliated gland cell
cmds: circular muscle of the digestive system
dcn: dorso-anterior commissure of the central neuropil
dfg: frontal gland ducts
dllm: dorsolateral longitudinal muscle
dlm: dorsal longitudinal muscle
e1-3: egg 1–3
ec: cili of an epidermis-cell
fpg1-2: FMRFamidergic perikarya of the postharyngeal ganglia 1 and 2
gd: opening of the frontal glands
go: glandular opening
gv: glandular vesicle
hg: hindgut
ksg: kidney-shaped gland cell
ladnc: lateral branch of the anterio-dorsal nerve cord
lavnc: lateral branch of the anterio-ventral nerve cord
lca: major caudal lobes
lci: minor caudal lobes
lg: lateral ganglion
llm: lateral longitudinal muscle
lmds: longitudinal muscle of the digestive system
ln: lateral nerve
lpn: lateral peripheral nerve
lpnp: lateral projection of the neuropil
lr: rostral lobe
lra: major rostral lobe
lrl: lateral rostral lobe
mg: male gonopore
mgg: male gonopore gland
mgp: perikaryon associated with the male gonopore
mlca: median nerve and connections from the dorsal commissure to the nerves of the major caudal lobes
mig: midgut
mnc: median nerve cord
mo: mouth opening
mrn: median rostral nerve
ne: epidermal nucleus
nlca: nerve of the major caudal lobe
nlci: nerve of the minor caudal lobe
nlra: nerve of the major rostral lobe
nlri: nerve of the lateral minor rostral lobe
nlrm: nerve of the median minor rostral lobe
nlrl: nerve of the lateral rostral lobe
np: neuropil
nrmg: nerve ring around the male gonopore
pcg: projection of the ciliated gland cell
pfg: posterior frontal gland
pg1-2: postpharyngeal ganglion 1–2
ph: pharynx
phg: pharyngeal gland
pln: peripheral longitudinal nerve
pp: posterior projection
ptg: projection of the tubular gland cell
rs: seminal receptacles
rsg: rod-shaped granules
sc: shortened cilium
snr: stomatogastric nerve ring
sp: perikarya of the stomatogastric nerve ring
spc: serotoninergic cell
spd: spermioduct
spf: sperm filaments
sph1-6: sphincter 1–6
ss: sensoria
ssm: star-shaped muscle
ssn: sickle-shaped nucleus
t: testis
tc: terminal commissure
tg: tubular glandl
tmr: transverse muscle ring complex
tpn: transverse ring of the peripheral nervous system
ts: tubulinergic sheath
tst: tubulinergic strands
vcn: ventral commissures of the neuropil
vllm: ventrolateral longitudinal muscle
vlm: ventral longitudinal muscle
vlnc: ventral longitudinal nerve cord
